# Diverse Gender and Sexual Identity in Romantic Partner Selection Experiences: An Exploration of Similarities, Differences, and Potential Explanations

**DOI:** 10.1007/s10508-025-03173-8

**Published:** 2025-07-15

**Authors:** Scott Devenport, Catriona Davis-McCabe, Barbara Mullan, Sam Winter

**Affiliations:** School of Population Health, Curtin University, Bentley, WA 6102 Australia

**Keywords:** Romantic relationships, Dating, Partner selection, Sexual identity, Sexuality/sexual orientation, Gender identity

## Abstract

The literature studying individuals’ selection of long-term romantic partners predominantly focuses on samples of majority gender and sexual identity (cisgender, heterosexual), leaving a gap in our knowledge regarding minority populations. In this study, 554 Australian adults seeking romantic partners (*M*_age_ = 32.06 years, 52.35% of whom reported minority gender and/or sexual identities) responded to an online survey regarding their partner selection experiences and beliefs, ideal and self-ratings on romantic partner traits, and self-perceived mate value. Factor analysis replicated established trait preference dimensions in this large, heterogeneous Australian sample, with dimensions remaining stable in majority and minority subsamples. Exploratory comparative and regression analyses generated preliminary insights into differences, similarities, interactions, and intersectional factors across identity groups. The commonly observed sex differences of men preferring aesthetic traits more than women, and women preferring empowered traits more than men were supported in comparative analyses. However, sex was not associated with preference for empowered traits in regression with identity, trait preferences, and self-perceived mate value. Self-perceived mate value findings replicated, challenged, and extended previous research, with asexual identity linked to lower self-perceptions. A preference for warm traits appeared to be a central factor through association with most other preferences and identities. As potential explanations, we discuss complex influences of evolutionary psychology, sociocultural factors, norms, and potential differences in partner selection processes. These findings contribute to our knowledge of partner selection experiences across majority and minority gender and sexual identity populations.

**Supplementary Information:**

The online version contains supplementary material available at 10.1007/s10508-025-03173-8.

## Introduction

Individual choice in romantic partner selection is inherently complex. Despite recent valuable contributions (Conroy-Beam et al., 2022; Eastwick et al., 2022), addressing this complexity remains challenging (for a review, see Devenport et al., 2023). Partner selection processes are inextricably linked to gender and sexual identity (van Anders, 2015), and the complexity that diverse identities present is frequently addressed by homogenizing samples (Csajbók & Berkics, 2017; Qian, 2022). The favored homogenous sample represents the statistical majority of individuals who identify as cisgender and heterosexual (Fletcher et al., 1999; Li et al., 2013).^1^ Frequent exclusion of those in the statistical minority results in relatively less knowledge about these populations (Wu et al., 2019). Despite the importance of gender and sexual identity in partner selection processes, the impact of identity and diversity on these processes is underexplored and leaves a gap in the literature.

### Romantic Partner Traits: Preference, Measurement, and Mate Value

Fletcher et al. (1999) established a partner selection research paradigm with their ideal standard model that focuses on how trait preferences are used to select partners based on how well ideals match the reality of prospective partners. The Fletcher and colleagues study also established the measurement of trait preference dimensions that capture ideal standards as constructs that individuals may use to select romantic partners (for reviews, see Campbell & Fletcher, 2015; Eastwick et al., 2014). Fletcher and colleagues identified three dimensions of trait preference, warmth–trustworthiness, vitality–attractiveness, and status–resources that researchers frequently use in their studies (Birkás et al., 2018; Travaglia et al., 2009; Valentine et al., 2020). Csajbók and Berkics (2017) observed seven trait preference dimensions of warmth, stability, appearance, passion, status, intellect, and dominance, leaving the matter of trait preference dimensionality open. There are mixed findings regarding trait preferences and the influence of ideal–actual comparisons on partner selection (Conroy-Beam et al., 2022; Eastwick et al., 2022). Furthermore, dimensions of trait preference have been validated mostly with majority gender and sexual identity samples; this highlights the need for further validation in minority samples, as there is no reason that the ideal standards model cannot be applied to the partner selection experiences of any population.

Researchers have linked trait preferences to an individual’s perception of their value as a romantic partner in short- and long-term contexts (Edlund & Sagarin, 2010, 2014). Csajbók and Berkics (2017) successfully predicted an individual’s self-perceived mate value with their self-ratings on trait dimensions. For male participants, higher self-perceived mate value was, in terms of explained variance, most strongly predicted by self-ratings of appearance, followed by passion, status, intellect, and dominance. For female participants, higher self-perceived mate value was most strongly predicted by self-ratings of appearance, followed by passion and dominance. Trait dimensions predicted 54% of the variance in self-perceived mate value for men and 45% for women. The large amount of explained variance indicates that perceptions of personal traits influence an individual’s self-image in romantic contexts—and what drives this influence differs slightly between men and women. As Csajbók and Berkics analyzed a majority identity sample, it is unclear if the findings apply across minority gender and sexual identities.

### Gender and Sexual Identity in Partner Selection Research

The exclusion of minority gender and sexual identities in partner selection research may preclude knowledge and insights attainable through observing differences and similarities between diverse identities (Qian, 2022; Wood & Brumbaugh, 2009). One reason for this exclusion may be the dominance of evolutionary psychological perspectives in the partner selection literature. While these frameworks are useful for examining broad factors, such as biological sex and cultural context, they often adopt a heteronormative in focus (Buss, 1989; Buss & Schmitt, 2019; Buss et al., 2020; Li & Meltzer, 2015). This heteronormative focus omits minority gender and sexual identities that are tied to an individual’s self-conceptualization and used to communicate information in intimate social interactions, such as dating (Arístegui et al., 2019; Hall et al., 2021; Kade, 2021; Van Kampen et al., 2024). While evidence suggests broader factors influence partner selection behaviors (Thomas et al., 2020), there is also evidence that narrow, individualized factors are also influential (Eastwick et al., 2023). While evolutionary perspectives may be suitable to explain some elements of partner selection in isolation (Park & van Leeuwen, 2015), social psychological, queer, and other perspectives could provide supplemental or alternative explanations for narrow, individualized factors. However, these perspectives remain underrepresented in the partner selection literature (Conley et al., 2011; Eastwick et al., 2023).

A core proposition of evolutionary psychological theories of partner selection is that men and women have different experiences due to having different evolutionary needs and outcomes (Trivers, 1972). For example, the chance that women could become pregnant and need to raise children has been suggested as an explanation for the higher preference women appear to have for men with higher status and more resources due to the support these traits would provide (Buss, 1989; Buss et al., 2020). Research findings have supported this idea, but studies are usually heteronormative and do not consider potential differences in experience that might arise from the diversity of gender and sexual identity (Thomas et al., 2020). Researchers have infrequently compared minority and majority sexual identity groups but sometimes support evolutionary perspectives with evidence of differences between men and women, regardless of sexual identity (Wood & Brumbaugh, 2009). However, other studies highlight the influence of factors specific to minority gender and sexual identity, such as a smaller pool of prospective partners and pressure from social norms that influence partner selection (Forde, 2011; Wu et al., 2019). There appears to be complexity in partner selection experiences arising from diverse gender and sexual identity, and investigating how more personal, interactive, and normative factors interact with broader concepts can assist in expanding our understanding (Mitchell & Knittel, 2023; Underwood, 2023; van Anders, 2015).

The shifts in research practices toward consideration of individuals with diverse identities are essential but introduce challenges (Hyde et al., 2019). As van Anders (2015) notes, partner selection contexts become even more complicated when certain identity classifications are “partnered,”—that is, they implicitly reference the gender or presence of prospective partners. For example, an individual identifying as bisexual may be interested in prospective partners who are assigned female at birth (henceforth, “female”) and those who are assigned male at birth (henceforth, “male”). Many bisexual individuals may indeed also be interested in binary transgender (i.e., trans men and women) or non-binary individuals (Blair & Hoskin, 2019), but the bisexual label leaves this interest unspecified. This lack of specification means the individual’s gender preferences cannot be assumed, which creates a problem for any research that classifies participants into discrete groups based on gender preferences, which has been a requirement of many partner selection studies.

Research that considers identity needs to be mindful of intersectionality: the intersections of multiple aspects of an individual’s identity that impact their lives (Cole, 2009; Crenshaw, 2017). Intersections usually take place across identity categories (Moore, 2012). However, sexual identity can be conceptualized as a superordinate category that contains identity subcategories, as most of these identities are related to realized or anticipated experiences with prospective partners (van Anders, 2015). For example, an individual could identify with a “sexual orientation identity,” such as gay or lesbian (i.e., attracted to the same assigned sex or gender identity as their own), a “sexual attraction identity,” such as asexual (i.e., experience little to no sexual attraction to partners, Vares, 2018), and a “partner number sexual identity,” such as polyamorous (i.e., prefer multiple intimate relationships, Obadia, 2020). Any of these identities could intersect with gender or other identities but can also intersect within sexual identity and potentially influence partner selection experiences specifically. This complexity compounds classification problems in quantitative research where grouping for comparison purposes may not permit individuals to be classified more than once per grouping (e.g., a “sexual identity” group, Williams et al., 2020). Researchers who employ quantitative designs that include people of minority sexual identities need to be aware of intersectionality and the consequent research complexities therein (Cole, 2009; Williams et al., 2020).

The disclosure of a minority sexual identity in partner selection contexts can lead to distinct, negative experiences (Bostwick & Hequembourg, 2014; Vares, 2018). For example, individuals who identify as asexual may be rejected by prospective partners upon disclosure that they feel little to no sexual attraction toward partners, an outcome that can lead to stress, poor mental health, and sometimes abandoning hope of ever finding a partner (Maxwell, 2017; Vares, 2018). A bisexual individual may be allosexual, meaning they experience sexual attraction toward partners, but prospective partners may still similarly reject them due to their bisexual identity and/or bisexual-specific phobia or “biphobia” (Bostwick & Hequembourg, 2014; Friedman et al., 2014). Disclosure of minority gender identity leads to similar rejection outcomes for trans people and can also lead to fetishization that also negatively impacts partner selection experiences (Albury et al., 2021; Griffiths & Armstrong, 2023). Despite potential similarities across identities (Wood & Brumbaugh, 2009), these findings suggest that adverse outcomes are a reality for those of minority gender and/or sexual identity, and therefore, it is important to investigate their experiences and any related consequences.

### The Current Study

More research that includes diverse gender and sexual identities is needed to investigate the nature of potential differences and similarities in partner selection experiences. As we have seen, minority gender and/or sexual identity populations are often intentionally excluded in partner selection studies (Csajbók & Berkics, 2017; Qian, 2022), and inclusive studies are somewhat rare (Arístegui et al., 2018; Carels et al., 2022; Ip et al., 2022). Trait preference studies are the most prominent form of investigation into partner selection experiences (Csajbók & Berkics, 2017; Fletcher et al., 1999) but require more validation in minority samples. Similarly, the applicability and dimensionality of traits and the utility of self-ratings to predict self-perceived mate value (Csajbók & Berkics, 2017) need to be explored in minority samples. Any research interested in identity needs to acknowledge intersectionality, especially when studies include minority samples (Cole, 2009; Crenshaw, 2017). Furthermore, the potential for individuals to identify with multiple sexual identities necessitates consideration of intersections within sexual identity.

Our exploratory study focuses on the partner selection experiences for individuals of majority and minority gender and sexual identities to address the gaps and limitations identified in the literature. We aimed to address the following research questions. First, what dimensions of trait preferences are suitable for a contemporary Australian sample? Second, are there any differences in trait preference dimensionality between majority and minority identity groups based on gender and sexual identity? Third, what are the similarities and differences in partner selection experiences, beliefs, preferences, and self-perceptions between majority and minority gender and sexual identities? Fourth, are there any interaction effects in potential differences between gender and sexual identities? Fifth, are there any indications of intersectional factors when considering partner selection experiences, beliefs, preferences, and self-perceptions? These questions aimed to explore what investigating similarities and differences across gender and sexual identity can tell us about partner selection experiences.

## Method

### Participants

We conducted recruitment online in 2021 through private dating-related social media groups, minority gender and/or sexual identity services or groups, and social media (Australian-specific Facebook and Reddit, and Instagram). Our recruitment selection criteria were Australian adults seeking a romantic partner(s), as those currently seeking partners would be closer to the experiences that questions asked about. While some partnered adults could answer some questions, others might find them inappropriate or difficult to answer. We calculated the minimum sample size through a priori power analyses using G*Power 3.1 (Faul et al., 2009), which indicated a minimum sample size of 318 would be necessary to detect small-to-medium effect sizes (Csajbók & Berkics, 2017; Eastwick & Finkel, 2008). This sample size also surpassed the minimum for factor analysis of 10 participants for each of the 27 trait preference items (Robinson, 2017).

Our recruitment methods resulted in 853 respondents with no unusual activity observed through Qualtrics survey bot detection. We removed one response for violating study selection criteria, three for responding uniformly, and 295 for having incomplete data for analysis. The removed cases were proportionate in terms of different demographics. The final sample size was 554, with an average age of 32.06 years (SD = 10.12) and no substantial age differences across gender and sexual identity groups. Participants were mostly of European ethnicities (76.90%), followed by Asian ethnicities (6.70%) and mixed ethnicities (4.20%). The remaining participant ethnicities were either missing or accounted for less than 1% of the sample.

Table 1 reports the assigned sex, gender identity, sexual identity, and minority–majority proportions for the whole sample. We have presented aspects of sexual identity according to the categories we discussed earlier: sexual orientation identity, sexual attraction identity, and partner number sexual identity. These terms do not imply any specific assigned sex or gender identity. “Different-attracted” comprises heterosexual participants, while “same-attracted” comprises gay and lesbian participants. “Single-attracted” comprises heterosexual, gay, and lesbian participants as these sexual identities all imply attraction to one type of prospective partner based on assigned sex or gender identity. “Multi-attracted” comprises bisexual and pansexual participants as these sexual identities imply attraction to multiple types of prospective partners based on assigned sex or gender identity.

**Table 1:** Demographic characteristics of the final sample (*N* = 554)

Characteristic	*n*	% of full sample
Assigned sex	–	–
Female	328	59.20%
Male	225	40.80%
Gender identity	–	–
Woman	291	52.52%
Man	218	39.35%
Non-binary	45	8.12%
Gender modality^a^	–	–
Cisgender	482	87.00%
Binary-trans	27	4.87%
Sexual identity	–	–
Sexual orientation identity	–	–
Different-attracted^b^	286	51.62%
Same-attracted^c^	99	17.87%
Multi-attracted	169	30.51%
Bisexual	115	20.76%
Pansexual	54	9.75%
Sexual attraction identity	–	–
Allosexual	516	93.14%
Asexual	38	6.86%
Partner number sexual identity	–	–
Monoamorous	516	93.14%
Polyamorous	38	6.86%
Identity classification	–	–
Majority	264	47.65%
Minority	290	52.35%

^a^Non-binary is not included in the gender modality classifications because we report it under gender identity. ^b^One non-binary participant (male) and three binary-trans participants (one trans woman and two trans men) identified as different-attracted. ^c^Three trans men participants identified as gay. Three trans women and 11 non-binary participants (all female) identified as lesbian. There is the same number of asexual and polyamorous participants, but only six participants identified as both

### Procedure

Participants responded to the Qualtrics online survey by clicking a link, reading an information sheet, and providing consent at the same time as confirming their adulthood and seeking of a romantic partner. The participants first answered questions regarding demographics, followed by questions on partner selection experiences and beliefs, trait preferences, and self-perceived mate value. Other questions followed these as part of a larger survey but did not concern partner selection experiences and are not relevant to the topic and analyses of this paper.

### Measures

#### Experience and Belief Questions

We used eight items to ask participants about their experiences and beliefs regarding aspects of the partner selection process. We developed these items to measure participants’ basic partner selection experiences and beliefs outside preference and self-perception. Participants’ responses were recorded on five-point Likert scales that ranged from 1, “strongly disagree,” to 5, “strongly agree.” Four items were asked about experiences, such as “It is challenging to find a romantic partner.” The remaining four items asked about beliefs; for example, “I believe in love at first sight.”

#### Prospective Partner Traits Measure

We gathered partner trait items from the literature (Boxer et al., 2013; Csajbók & Berkics, 2017; Fletcher et al., 1999; Hill, 1945) and then reviewed these items for distinctiveness, relevance, and suitability to a contemporary Australian sample. This review and refinement occurred over two group consultations with key informants of majority and minority sexual, gender, and cultural identities. We finalized a set of 27 items after consultation and subsequent minor refinements. Participants rated the importance of these items on five-point Likert scales, first about an ideal partner, and then in describing themselves; their responses ranged from 1, “not at all important/descriptive,” to 5, “extremely important/descriptive.” After factor analysis, we averaged the responses of items loading together to represent preferences and self-ratings for each partner trait dimension.

#### Mate Value Scale

Edlund and Sagarin (2014) developed the Mate Value Scale to measure how an individual perceives their own value as a romantic partner. Four seven-point Likert scale items measure the self-perceived mate value of a participant; for example, “Overall, how would you rate your level of desirability as a partner on the following scale?” The four responses are averaged to calculate each participant’s self-perceived mate value measurement. Previous research has found that the Mate Value Scale possesses adequate validity and strong internal consistency (*α* = 0.81 to 0.89; Edlund & Sagarin, 2014; Gillen et al., 2016), which we similarly found in the current study (*α* = 0.87).

### Data Analysis

Due to potentially complex intersections between aspects of identity, we employed multiple analyses to capture as much of this complexity as possible (Williams et al., 2020). We used bivariate correlations to explore relationships between test variables (i.e., experience and belief questions, self-perceived mate value, ideal ratings, and self-ratings on trait dimensions) and dichotomous identity groups. We used factorial ANOVA analyses to compare assigned sex and sexual orientation identity groups on test variables while also testing for any interaction effects. We also used a one-way between-group ANOVA to compare gender identity groups to enable comparison of these findings to those when comparing assigned sex.

We used multiple regression analyses to capture the complexity and potential intersectional factors by placing gender and sexual identities within the same regression model. The criteria for the first set of regression analyses were each test variable, apart from self-perceived mate value, which was a predictor in the first regression set and the criterion for a second set of regression analyses. The predictors for the first set of regression analyses were assigned sex, gender identities, sexual identities, ideal ratings, and self-perceived mate value. We used self-perceived mate value as a proxy for self-ratings. These variables were strongly correlated in our data and previous research (Csajbók & Berkics, 2017), and self-ratings were multicollinear with ideal ratings. The predictors for the second set of regression analyses included assigned sex, gender identities, sexual identities, and trait self-ratings. We also predicted self-perceived mate value for each gender and sexual identity subsample while considering statistical power issues and the potential for minority identity subsamples to be small when acting as predictors within other minority identity subsamples.

## Results

Five of the 554 retained responses did not provide self-perceived mate value data, leaving 549 participants in analyses involving self-perceived mate value. Partner trait questions had some missing data, but this was missing completely at random (Little’s MCAR *p* = .28). We used expectation maximization to replace these missing values and reviewed for errors (Wu, 1983). Data were otherwise complete.

### Prospective Partner Trait Factor Analyses

We confirmed that factor analysis assumptions, including sampling adequacy and multicollinearity, were met, except for minor normality violations against which factor analysis is robust. Partner trait items were standardized in the ideal and self-rating contexts to control for spurious correlations between items (Csajbók & Berkics, 2017) and counter-normative desirability effects (Wood & Furr, 2016). We employed principal axis factoring with Promax rotation to analyze the dimensional structure of ideal, self-, and combined standardized ratings. The large sample size also permitted an exploration of dimensionality in majority and minority subsamples.

Our initial eigenvalue extraction of combined standardized ratings items resulted in a 6-dimension solution that accounted for 45.07% of trait rating variance, but the item “affectionate” was cross-loaded. Removing “affectionate” caused other traits to unload or cross-load. A seventh dimension had an eigenvalue of 0.99; other analyses resulted in seven-dimension solutions. We, therefore, forced a seven-dimension solution onto the combined standardized ratings that accounted for 47.64% of trait rating variance and resolved the “affectionate” cross-loading but unloaded “good communicator” and “shared values” items. We retained the seven-dimension solution as it accounted for a large amount of variance; items that did not load were the same as alternative solutions, and we judged it to be parsimonious.

Our observed seven-dimension model mostly aligns with Csajbók and Berkics’ (2017) set, where they observed an eighth dimension that included humor but could not reconcile it due to cross-loading issues. The easy-going dimension had low internal consistency, but we retained it as humor-related partner traits are a consistent and important feature in partner selection literature (Brown et al., 2023; Hall, 2015). The intimate dimension also had low internal consistency, but we retained it due to alignment with Csajbók and Berkics’ passionate dimension. We also forced a three-factor model on the data to test Fletcher et al.’s (1999) original dimensionality. After resolving issues with cross-loading, the best model required the removal of six items and accounted for 37.68% of the variance. The dimensions of this model approximated Fletcher et al.’s set, but we retained the seven-dimension model as it accounted for more variance and retained more items.

Confirmatory factor analyses supported the suitability of the seven-dimension model despite two-item factors and after accounting for appropriate co-variances between items within the empowered and reliable factors (Bollen, 1989), *χ*^2^(247) = 824.57, RMSEA = 0.065 (0.060–0.070), CFI = 0.867, TLI = 0.838, SRMR = 0.040. Measurement invariance analyses comparing the seven-dimension model in majority and minority subsamples supported configural invariance (*χ*^2^[494] = 1140.94, RMSEA = 0.049 [0.045–0.052], CFI = 0.853, TLI = 0.821, SRMR = 0.068) and metric invariance (*p* = .617), indicating that the model was invariant across these groups (Collier, 2020). While there were some issues with the model, including unloaded items and items that were expressed poorly and could have been difficult to respond to (e.g., “sexual compatibility”), the seven dimensions aligned with previous literature (Csajbók & Berkics, 2017) and were suitable for analysis. We report the dimension names, item loadings, internal consistency by context, and variance accounted for by each dimension in the seven-dimension model in Table 2.

**Table 2:** Rotated factor matrix for the seven-dimension extraction of combined ideal and self-ratings on partner traits measure (*N* = 554)

Romantic trait dimension Variance accounted for	Warm 21.44%	Empowered 9.29%	Smart 5.05%	Dependable 4.10%	Aesthetic 3.78%	Intimate 2.06%	Easy-going 1.92%
Considerate	**.88**	−.11	.06	.04	.12	−.10	.01
Compassionate	**.86**	−.04	−.05	.00	.01	.13	−.08
Supportive	**.62**	.09	.02	.08	−.10	.08	−.07
Open-minded	**.56**	.05	.01	−.07	−.03	−.01	.15
Patient	**.47**	−.06	.04	.05	.05	−.05	.10
Driven	−.03	**.76**	.01	.02	−.02	.00	−.27
Assertive	−.08	**.65**	.03	−.07	−.09	.13	.18
Outgoing	−.02	**.64**	−.16	−.13	.04	.18	.14
Independent	.13	**.54**	−.01	.03	−.03	−.26	.19
Resilient	.21	**.51**	.05	.00	−.04	−.13	.18
Financial stability	−.26	**.49**	.15	.21	.08	−.08	−.15
Self-confident	−.12	**.48**	.06	−.03	.17	.09	.24
Intelligent	−.07	−.14	**.96**	.00	−.03	.05	.07
Knowledgeable	.18	.12	**.61**	−.10	−.03	.01	−.01
Well-educated	.07	.19	**.51**	−.02	.08	−.01	−.24
Trustworthy	.03	−.06	−.03	**.87**	−.03	.01	.09
Loyal	.03	−.04	−.02	**.75**	−.02	.08	.07
Reliable	.21	.29	−.09	**.41**	.01	.03	.01
Good looking	.09	−.08	.03	−.02	**.85**	.03	.03
Good body	−.01	.06	−.05	−.03	**.78**	−.02	.02
Affectionate	.09	−.05	.02	.02	−.07	**.77**	−.08
Sexually compatible	−.16	−.08	.08	.17	.17	**.52**	.09
Passionate	.26	.15	−.03	−.09	.04	**.38**	.00
Sense of humor	.08	.10	.17	.10	−.06	.06	**.40**
Laid back	.04	.09	−.13	.08	.07	−.05	**.38**
Good communicator^a^							
Shared values^a^							
Ideal rating Cronbach’s α	.72	.75	.72	.64	.81	.59	.36
Self-rating Cronbach’s α	.75	.73	.74	.74	.76	.55	.26

Bold figures indicate primary factor loadings

Promax rotation used. ^a^ Item did not load on to any dimension. Split-half reliability was similar for aesthetic and easy-going dimensions

### Bivariate, Factorial, and Interaction Analyses Across Assigned Sex and Sexual Orientation Groups

We checked the assumptions for each analysis, and most test variables had normal distributions. Ideal and self-ratings of dependability, beliefs about “leagues” and “love at first sight,” and all experience questions were skewed toward higher measurements. We note that these skewed responses could indicate that, in this sample, dependability traits are highly important in prospective partners and strongly self-perceived. Similarly, beliefs about “leagues” and “love at first sight” seem to be commonly held by participants and experiences of being challenged by the tasks of finding, choosing, quickly deciding on, and thinking a great deal about a romantic partner were common. We observed homogeneity of variance across test variables, with only slight heterogeneity in the experience of quick decisions on prospective partners, beliefs about ‘leagues’ and fluid sexuality, and aesthetic ideal ratings. We report the bivariate correlations between test variables in Table 3.

**Table 3:** Bivariate correlations and descriptive statistics for test variables (*N* = 554; *N* = 549 for self-perceived mate value)

	1	2	3	4	5	6	7	8	9						
1. It is challenging to find a romantic partner	–														
2. It is challenging to choose a romantic partner	.22⁂	–													
3. I quickly decide if a person is suitable as a potential romantic partner	−.01	−.11*	–												
4. I put a lot of thought into selecting a romantic partner	.09*	.20⁂	.09*	–											
5. I believe that people are in different 'leagues'	.18⁂	.06	.13⁑	−.01	–										
6. I believe that people sometimes 'settle' for a romantic partner	.01	−.01	.03	.03	.06	–									
7. I believe in ‘love at first sight’	.06	−.01	.10*	−.01	.10*	−.02	–								
8. I believe that sexuality is fluid	−.07	−.07	−.06	.00	−.08	−.00	.01	–							
9. Self-perceived mate value	−.27⁂	−.06	.20⁂	.08	−.14⁑	.01	.06	.02	–						
10. Ideal-Warm	.00	.07	−.09*	.10*	.10*	.06	−.04	.25⁂	.08						
11. Ideal-Empowered	−.09*	.07	.09*	.11*	−.06	.04	.06	.02	.26⁂						
12. Ideal-Smart	.04	.04	.14*	.08	−.04	.15⁑	−.04	.06	.13⁑						
13. Ideal-Dependable	.06	−.01	.08*	.11*	.03	.03	.07	−.05	.11*						
14. Ideal-Aesthetic	−.05	.01	.14⁂	.02	.09*	.04	.10*	−.18	.19⁂						
15. Ideal-Intimate	−.01	.00	.07	.00	.03	.12⁑	.13⁑	−.01	.18⁂						
16. Ideal-Easy-going	−.07	.00	.01	.01	−.00	.08*	.04	.02	.06						
17. Self-Warm	.02	.05	−.11*	.16⁂	−.13⁑	.04	.03	.17⁂	.13⁑						
18. Self-Empowered	−.17⁂	.03	.12⁑	.10*	−.10*	.11⁑	−.00	−.06	.46⁂						
19. Self-Smart	.04	−.03	.06	.07	−.04	.13⁑	−.03	.01	.20⁂						
20. Self-Dependable	.08	.03	.07	.12⁑	−.01	.03	.00	−.04	.08						
21. Self-Aesthetic	−.21⁂	.02	.17⁂	.05	−.06	−.02	.08	−.07	.56⁂						
22. Self-Intimate	−.03	−.02	.08	.03	.01	.06	.14⁂	.00	.30⁂						
23. Self-Easy-going	−.04	.05	.08	−.01	.02	.04	.11⁑	−.07	.10*						

	10	11	12	13	14	15	16	17	18	19	20	21	22	23	Mean (SD)
1															3.94 (1.10)
2															3.37 (1.07)
3															3.60 (1.14)
4															4.11 (1.00)
5															3.49 (1.24)
6															4.29 (0.73)
7															2.73 (1.21)
8															3.75 (1.19)
9															4.79 (1.15)
10	–														4.14 (0.53)
11	.29⁂	–													3.24 (0.60)
12	.24⁂	.45⁂	–												3.37 (0.75)
13	.47⁂	.33⁂	.21⁂	–											4.39 (0.53)
14	−.12⁑	.17⁂	.13⁑	.08	–										2.87 (0.84)
15	.25⁂	.28⁂	.19⁂	.32⁂	.29⁂	–									3.92 (0.67)
16	.23⁂	.29⁂	.14⁂	.18⁂	.05	.19⁂	–								3.68 (0.64)
17	.56⁂	.15⁂	.14⁂	.27⁂	−.13⁑	.24⁂	.20⁂	–							4.03 (0.61)
18	.13⁑	.51⁂	.22⁂	.21⁂	.18⁂	.20⁂	.22⁂	.24⁂	–						3.38 (0.65)
19	.17⁂	.08	.40⁂	.11⁑	.08	.11⁑	.04	.20⁂	.34⁂	–					3.82 (0.66)
20	.33⁂	.23⁂	.16⁂	.57⁂	−.07	.16⁂	.16⁂	.43⁂	.30⁂	.13⁑	–				4.30 (0.65)
21	.02	.25⁂	.16⁂	.07	.41⁂	.22⁂	.09*	−.03	.36⁂	.22⁂	.01	–			2.67 (0.83)
22	.22⁂	.23⁂	.12⁂	.25⁂	.17⁂	.69⁂	.14⁑	.42⁂	.30⁂	.18⁂	.23⁂	.25⁂	–		3.67 (0.75)
23	.00	.07	−.02	.11⁑	.09*	.15⁂	.47⁂	.22⁂	.24⁂	.07	.16⁂	.07	.23⁂	–	3.60 (0.75)

*p < .05, ⁑ *p* < .01, ⁂ *p* < .001, two-tailed. SD = standard deviation. Self-perceived mate value measured on a 7-point Likert scale; all other variables measured on 5-point Likert scales

We report the bivariate correlations between test variables and dichotomous identity groups in Table 4. We observed some significant correlations for dichotomous identity groups not presented in Table 4. Binary-trans and non-binary (coded 0 and 1, respectively) identity was significantly related to dependable ideal and self-ratings (*r*[70] = −.28 to −.30, *p* = .011 to .018), and belief in love at first sight (*r*[70] =  −.23, *p* = .049). Bisexual and pansexual (coded 0 and 1, respectively) identity was significantly related to belief about “leagues,” aesthetic and intimate ideal ratings (all, *r*[149] = −.18, *p* = .026 to .030), and belief that people ‘settle’ (*r*[149] = -.24, *p* = .003). Different-attracted and same-attracted (coded 0 and 1, respectively) identity was significantly related to finding a partner being challenging, beliefs about sexuality being fluid, and warm and intimate ideal and self-ratings (*r*[383] = .12 to .13, *p* = .012 to .022).

**Table 4:** Bivariate correlations of test variables with dichotomous-coded gender and sexual identity groups (*N* = 554; *N* = 549 for self-perceived mate value)

	MALE–FEMALE	GEND-MW	GEND-BINAR	CIS- TGD	SINGLE-MULTI	HET-NOT	ALLO-ASEX	MONO-POLY	MAJO-MINO
It is challenging to find a romantic partner	−.06	−.07	.05	.07	−.01	.07	.07	.01	.09*
It is challenging to choose a romantic partner	.08	.07	−.01	.01	.04	.04	.06	.02	.05
I quickly decide if a person is suitable as a potential romantic partner	.07	.08	−.06	−.11*	−.07	−.10*	−.13⁑	−.09*	−.12⁑
I put a lot of thought into selecting a romantic partner	.04	.06	.07	.07	.02	.02	.05	−.00	−.00
I believe that people are in different 'leagues'	−.08	−.08	−.13⁑	−.13⁑	−.08	−.08	−.02	−.13⁑	−.09*
I believe that people sometimes 'settle' for a romantic partner	.06	.05	−.03	−.05	.04	.02	.01	.03	.05
I believe in ‘love at first sight’	−.13⁑	−.09*	−.10*	−.06	−.12⁑	−.02	−.06	−.05	−.02
I believe that sexuality is fluid	.25⁂	.23⁂	.15⁂	.18⁂	.37⁂	.34⁂	.05	.15⁂	.33⁂
Self-perceived mate value	.15⁂	.13⁑	−.06	−.10*	−.10*	−.09*	−.22⁂	.01	−.11⁑
Ideal-Warm	.32⁂	.29⁂	.08	.13⁑	.21⁂	.22⁂	.08	.10*	.20⁂
Ideal-Empowered	.17⁂	.22⁂	−.04	−.08	−.10*	−.09*	−.10*	−.06	−.10*
Ideal-Smart	.18⁂	.18⁂	−.01	−.05	−.01	−.04	−.03	−.08*	−.06
Ideal-Dependable	.17⁂	.18⁂	−.13⁑	−.07	−.10	−.07	−.06	−.14⁂	−.09*
Ideal-Aesthetic	−.22⁂	−.22⁂	−.11⁑	−.15⁑	−.22⁂	−.21⁂	−.13⁑	−.09	−.22⁂
Ideal-Intimate	−.02	−.02	−.05	−.06	−.05	.03	−.21⁂	.06	.00
Ideal-Easy-going	−.01	.02	.02	−.05	−.05	−.06	−.04	−.03	−.05
Self-Warm	.15⁂	.13⁑	.09*	.11*	.14⁑	.17⁂	.06	.09*	.14⁂
Self-Empowered	.02	.04	−.06	−.11*	−.14⁂	−.11*	−.14⁑	−.07	−.11*
Self-Smart	.02	.02	−.01	−.01	.00	.05	−.04	−.03	.04
Self-Dependable	.09	.11*	−.08	−.03	−.06	−.06	−.01	−.09*	−.06
Self-Aesthetic	.01	.01	−.04	−.05	−.09*	−.11*	−.12⁑	−.06	−.11*
Self-Intimate	−.01	−.03	.01	−.00	−.00	.06	−.19⁂	.10*	.02
Self-Easy-going	−.25⁂	−.23⁂	−.07	−.11*	−.12⁑	−.12⁑	−.06	−.03	−.10*

*p < .05, ⁑ *p* < .01, ⁂ *p* < .001, two-tailed. Female/woman and minority groups are coded as 1. MALE–FEMALE refers to assigned sex

GEND-MW = gender: men and women. GEND-BINAR = gender: binary and non-binary. CIS-TGD = cisgender and trans/gender diverse

SINGLE-MULTI = single-attracted and multi-attracted. HET-NOT = heterosexual and not heterosexual. ALLO-ASEX = allosexual and asexual

MONO-POLY = monoamorous and polyamorous. MAJO-MINO = majority and minority

We report our factorial ANOVA analyses that compared assigned sex and sexual orientation identity groups on each test variable in Table 5. Asexual, polyamorous, and non-binary groups were too small for factorial analysis. We did not adjust alpha levels for any analysis as such a procedure may be unnecessary without a disjunctive hypothesis (García-Pérez, 2023; Rubin, 2021). However, we report degrees of significance to allow for interpretation with adjustment. Our results were not substantially or significantly impacted by standardizing measurements to control for normative desirability effects (Eastwick et al., 2022; Wood & Furr, 2016). The results of the gender identity ANOVA matched those observed across assigned sex and in bivariate correlations. However, we note that small numbers of binary-trans participants may have made differentiating between gender identity and assigned sex difficult, potentially obscuring findings.

**Table 5:** Factorial ANOVA results for assigned sex, sexual orientation identity groups, and interactions (*N* = 554; *N* = 549 for self-perceived mate value)

	Assigned sex		Sexual orientation identity			Interaction *η*^2^
	Male	Female	Different	Same	Multi	
	*M* (SD)	*M* (SD)	*M* (SD)	*M* (SD)	*M* (SD)	
	Main effect *η*^2^		Main effect* η*^2^			
It is challenging to find a romantic partner	4.03 (1.08)	3.88 (1.11)	3.86 (1.11)^a^	4.19 (1.05)^a^	3.93 (1.09)	.00
	.00		.01*			
It is challenging to choose a romantic partner	3.63 (1.07)	3.81 (1.07)	3.69 (1.07)	3.76 (1.07)	3.80 (1.07)	.00
	.01		.00			
I quickly decide if a person is suitable as a potential romantic partner	3.51 (1.12)	3.67 (1.15)	3.71 (1.05)	3.51 (1.20)	3.48 (1.24)	.00
	.00		.01			
I put a lot of thought into selecting a romantic partner	4.06 (1.07)	4.15 (0.95)	4.09 (0.97)	4.11 (1.06)	4.14 (1.02)	.00
	.00		.00			
I believe that people are in different 'leagues'	3.60 (1.21)	3.41 (1.26)	3.58 (1.17)	3.45 (1.33)	3.34 (1.29)	.01
	.01*		.01			
I believe that people sometimes 'settle' for a romantic partner	4.23 (0.74)	4.32 (0.71)	4.27 (0.77)	4.25 (0.69)	4.33 (0.66)	.01*
	.01*		.00			
I believe in ‘love at first sight’	2.91 (1.25)	2.60 (1.16)	2.75 (1.16)	3.01 (1.20)	2.51 (1.24)	.01
	.02⁑		.01			
I believe that sexuality is fluid	3.39 (1.27)	3.99 (1.07)	3.36 (1.21)^ab^	3.73 (1.21)^a^	4.41 (0.80)^b^	.01*
	.03⁂		.14⁂			
Self-perceived mate value	4.59 (1.18)	4.94 (1.12)	4.89 (1.10)^a^	4.81 (1.06)^b^	4.62 (1.28)^ab^	.00
	.02⁂		.02*			
Ideal-Warm	3.94 (0.53)	4.28 (0.48)	4.03 (0.55)^ab^	4.18 (0.51)^a^	4.31 (0.46)^b^	.00
	.09⁂		.04⁂			
Ideal-Empowered	3.12 (0.63)	3.33 (0.57)	3.30 (0.60)^a^	3.24 (0.60)^b^	3.16 (0.59)^ab^	.01
	.02⁂		.02*			
Ideal-Smart	4.28 (0.53)	3.48 (0.73)	3.40 (0.76)	3.29 (0.73)	3.36 (0.75)	.01*
	.04⁂		.00			
Ideal-Dependable	3.10 (0.89)	4.47 (0.52)	4.43 (0.52)^a^	4.42 (0.47)^b^	4.31 (0.58)^ab^	.00
	.03⁂		.02*			
Ideal-Aesthetic	3.93 (0.70)	2.72 (0.78)	3.04 (0.83)^ab^	2.86 (0.83)^a^	2.59 (0.81)^b^	.00
	.03⁂		.04⁂			
Ideal-Intimate	3.93 (0.70)	3.91 (0.65)	3.90 (0.68)	4.07 (0.58)	3.86 (0.70)	.00
	.00		.01			
Ideal-Easy-going	3.68 (0.65)	3.68 (0.64)	3.72 (0.63)	3.66 (0.68)	3.63 (0.64)	.00
	.00		.00			
Self-Warm	3.92 (0.61)	4.10 (0.60)	3.93 (0.62)^ab^	4.09 (0.59)^a^	4.15 (0.59)^b^	.00
	.02⁑		.03⁂			
Self-Empowered	3.36 (0.65)	3.39 (0.65)	3.45 (0.62)^a^	3.43 (0.65)^b^	3.24 (0.68)^ab^	.00
	.00		.02⁑			
Self-Smart	3.81 (0.71)	3.83 (0.60)	3.79 (0.65)	3.91 (0.64)	3.83 (0.70)	.01
	.00		.01			
Self-Dependable	4.23 (0.71)	4.35 (0.60)	4.34 (0.66)	4.29 (0.64)	4.24 (0.63)	.00
	.01*		.00			
Self-Aesthetic	2.66 (0.83)	2.68 (0.82)	2.76 (0.80)^a^	2.61 (0.85)	2.57 (0.84)^a^	.01
	.00		.01*			
Self-Intimate	3.69 (0.75)	3.67 (0.75)	3.63 (0.73)	3.81 (0.73)	3.67 (0.80)	.00
	.00		.01			
Self-Easy-going	3.82 (0.72)	3.45 (0.74)	3.69 (0.75)	3.58 (0.69)	3.46 (0.78)	.02⁑
	.03⁂		.01			

*p < .05, ⁑ *p* < .01, ⁂ *p* < .001, two-tailed. Different = different-attracted (heterosexual). Same = same-attracted (gay, lesbian). Multi-attracted (bisexual, pansexual). *M* = mean. SD = standard deviation. Self-perceived mate value measured on a 7-point Likert scale; all other variables measured on 5-point Likert scales. Means are compared within each row, and those with the same letter superscript within the same row are significantly different

We note 10 non-significant and 13 significant comparisons across the assigned sex comparisons. Most differences were of small effect size, except a higher preference for warm partners in female participants. We observed the same number of significant and non-significant comparisons across sexual orientation identity comparisons. Differences were again of small effect size, except for beliefs about sexuality being fluid, which female participants and those who were of minority gender and sexual orientation identity more strongly believed. Importantly, the pattern of test variables that participants significantly differed on was not the same when comparing assigned sex and sexual orientation identity groups. This difference in pattern could indicate that assigned sex and sexual orientation identity provide unique contributions to various partner selection experiences, beliefs, preferences, and self-perceptions.

We observed four significant interaction effects in the assigned sex and sexual orientation identity factorial ANOVA. First, female participants had stronger beliefs that people settle for partners, but not in the different-attracted group. Second, male participants had weaker beliefs about sexuality being fluid with medium effect size, but not in the multi-attracted group. Third, female participants preferred smart partners more only within the same-attracted group. Fourth, male participants rated themselves as more easy-going, but not within the same-attracted group. There were no unique interaction effects when replacing sexual orientation identity groups with single- and multi-attracted groups.

**Fig. 1:**
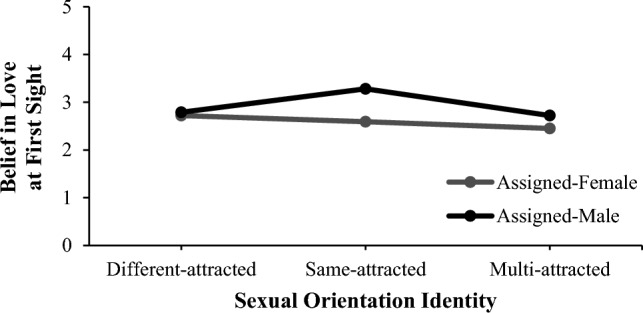
Significant interaction effect between assigned sex and sexual orientation identity on belief in love at first sight. *Note* This interaction effect was significant only when we combined same-attracted and multi-attracted groups into a minority sexual orientation identity group. However, this perspective illustrates the reasons behind the interaction more clearly

**Fig. 2:**
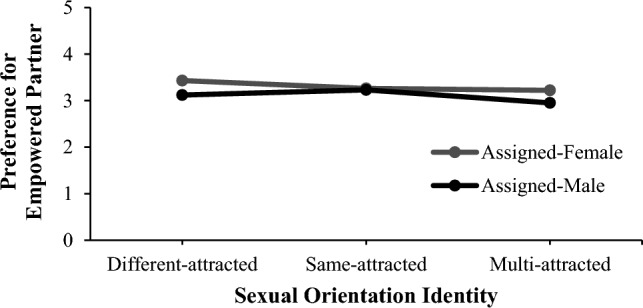
Significant interaction effect between assigned sex and sexual orientation identity on preference for empowered partners. *Note* This interaction effect was significant only when we combined same-attracted and multi-attracted groups into a minority sexual orientation identity group. However, this graph illustrates the reasons behind the interaction more clearly

When we grouped sexual identity groups by majority and minority sexual identities (different-attracted vs. same- and multi-attracted), we observed two further significant interaction effects with small effect sizes (*η*^2^ = 0.01). First, male participants had significantly stronger belief in love at first sight within the same-multi combined group only. Male, same-attracted participants who held stronger beliefs than participants of any other assigned sex or sexual orientation identity appear to drive this difference; see Fig. 1 for a visual representation of this interaction from the sexual orientation identity perspective. Second, female participants preferred empowered partners significantly more in the different-attracted group only. This may be due to female participants having a higher preference in the multi-attracted group but not in the same-attracted group; see Fig. 2 for a visual representation of this interaction from the sexual orientation identity perspective.

### Multiple Regression Analyses Predicting Partner Selection Experiences, Beliefs, Trait Preferences, and Trait Self-Perceptions

In Table 6, we present a selection of multiple regression analyses predicting all test variables pertinent to this paper’s discussion and highlight broad trends in our findings. We made this decision to focus our reporting and discussion; a complete set of these analyses is available in Appendix A. The predictive utility of age, a combination of binary-trans and non-binary predictors, and alternative majority and minority identity predictors were each explored. However, none significantly or substantially impacted any of the analyses. When we separated the multi-attracted predictor into bisexual and pansexual predictors, there was a minor increase in predictive utility for intimate and aesthetic ideal ratings, as bisexual participants more strongly preferred these traits. Our model significantly predicted belief in love at first sight, but there were no significant individual predictors. We evaluated a model employing only those variables correlating with this belief (see Table 4). We found that intimate ideal ratings and assigned sex were distinct significant predictors (adjusted *R*^2^ = .21, *p* < .001, both predictors *sr*^2^ = .01).

**Table 6:** Selected multiple regression analysis results predicting test variables with gender and sexual identity, partner trait ideal ratings, and self-perceived mate value (*N* = 549)

	*β*	*β* 95% CI		*sr*^2^		*β*	*β* 95% CI		*sr*^2^
		Low	High				Low	High	
It is challenging to find a romantic partner (Adj. *R*^2^ = .09)					I quickly decide if a person is suitable as a potential romantic partner (Adj. *R*^2^ = .08)				
Female	−.03	−.12	.06	.00	Female	.09	−.01	.18	.01
Binary-trans	.03	−.06	.11	.00	Binary-trans	−.05	−.13	.03	.00
Non-binary	.04	−.05	.13	.00	Non-binary	.00	−.00	.00	.00
Same-attracted	.09*	.00	.18	.01	Same-attracted	−.02	−.11	.13	.00
Multi-attracted	−.02	−.12	.08	.00	Multi-attracted	.01	−.09	.11	.00
Asexual	.01	−.08	.10	.00	Asexual	−.07	−.16	.01	.00
Polyamorous	.03	−.06	.12	.00	Polyamorous	−.03	−.11	.06	.00
Ideal-Warm	−.03	−.13	.08	.00	Ideal-Warm	−.18⁂	−.29	−.08	.02
Ideal-Empowered	−.08	−.18	.02	.00	Ideal-Empowered	−.04	−.14	.06	.00
Ideal-Smart	.11*	.02	.20	.01	Ideal-Smart	.13⁑	.03	.22	.01
Ideal-Dependable	.12*	.02	.22	.01	Ideal-Dependable	.11*	.01	.21	.01
Ideal-Aesthetic	−.01	−.10	.08	.00	Ideal-Aesthetic	.07	−.02	.16	.00
Ideal-Intimate	.02	−.08	.11	.00	Ideal-Intimate	.01	−.08	.10	.00
Ideal-Easy-going	−.06	−.15	.03	.00	Ideal-Easy-going	.00	−.08	.08	.00
Self-Mate-Value	−.26⁂	−.35	−.17	.06	Self-Mate-Value	.15⁂	.06	.24	.02
I believe that sexual identity is fluid (Adj. *R*^2^ = .20)					Ideal-Warm (Adj. *R*^2^ = .40)				
Female	.15⁑	.06	.23	.02	Female	.17⁂	.09	.24	.02
Binary-trans	.03	−.05	.11	.00	Binary-trans	.07*	.00	.14	.00
Non-binary	.00	−.10	.10	.00	Non-binary	.01	−.06	.08	.00
Same-attracted	.12⁑	.04	.22	.01	Same-attracted	.12⁑	.05	.19	.01
Multi-attracted	.30⁂	.21	.39	.06	Multi-attracted	.19⁂	.12	.27	.03
Asexual	−.03	−.11	.05	.00	Asexual	.08*	.01	.14	.01
Polyamorous	.05	−.03	.13	.00	Polyamorous	.10⁑	.03	.17	.01
Ideal-Warm	.16⁑	.07	.26	.02	Ideal-Warm	–	–	–	–
Ideal-Empowered	−.01	−.11	.09	.00	Ideal-Empowered	.08	.00	.16	.00
Ideal-Smart	.05	−.04	.13	.00	Ideal-Smart	.09*	.01	.16	.01
Ideal-Dependable	−.13⁑	−.23	−.04	.01	Ideal-Dependable	.37⁂	.30	.45	.11
Ideal-Aesthetic	−.06	−.15	.02	.00	Ideal-Aesthetic	−.12⁑	−.19	−.04	.01
Ideal-Intimate	−.01	−.11	.08	.00	Ideal-Intimate	.12⁑	.04	.19	.01
Ideal-Easy-going	.02	−.06	.10	.00	Ideal-Easy-going	.14⁂	.07	.21	.02
Self-Mate-Value	.03	−.05	.11	.00	Self-Mate-Value	.02	−.05	.08	.00
Ideal-Empowered (Adj. *R*^2^ = .33)					Ideal-Smart (Adj. *R*^2^ = .22)				
Female	.08	−.00	.15	.00	Female	.08	−.00	.17	.01
Binary-trans	−.01	−.08	.06	.00	Binary-trans	−.03	−.11	.05	.00
Non-binary	.01	−.07	.08	.00	Non-binary	.00	−.11	.11	.00
Same-attracted	.00	−.04	.04	.00	Same-attracted	−.04	−.09	.03	.00
Multi-attracted	−.07	−.15	.01	.00	Multi-attracted	.01	−.08	.10	.00
Asexual	−.02	−.10	.05	.00	Asexual	.01	−.07	.09	.00
Polyamorous	−.01	−.08	.07	.00	Polyamorous	−.08	−.16	.00	.01
Ideal-Warm	.08	−.01	.17	.00	Ideal-Warm	.11*	.02	.21	.01
Ideal-Empowered	–	–	–	–	Ideal-Empowered	.38⁂	.29	.47	.11
Ideal-Smart	.32⁂	.25	.40	.09	Ideal-Smart	–	–	–	–
Ideal-Dependable	.13⁑	.05	.22	.01	Ideal-Dependable	.00	−.10	.09	.00
Ideal-Aesthetic	.07	−.01	.14	.00	Ideal-Aesthetic	.09*	.00	.17	.01
Ideal-Intimate	.07	−.01	.15	.00	Ideal-Intimate	.04	−.04	.13	.00
Ideal-Easy-going	.17⁂	.10	.24	.03	Ideal-Easy-going	−.01	−.09	.07	.00
Self-Mate-Value	.14⁂	.07	.21	.02	Self-Mate-Value	−.02	−.09	.06	.00
Ideal-Dependable (Adj. *R*^2^ = .34)					Ideal-Aesthetic (Adj. *R*^2^ = .21)				
Female	.08	−.00	.16	.01	Female	−.22⁂	−.31	−.14	.01
Binary-trans	.06	−.01	.13	.00	Binary-trans	−.03	−.11	.05	.00
Non-binary	−.08*	−.16	−.01	.00	Non-binary	.01	−.07	.10	.00
Same-attracted	−.05	−.06	.02	.00	Same-attracted	−.11⁑	−.15	−.02	.01
Multi-attracted	−.15⁂	−.23	−.07	.00	Multi-attracted	−.13⁑	−.22	−.04	.01
Asexual	.00	−.06	.07	.00	Asexual	.01	−.07	.09	.00
Polyamorous	−.13⁂	−.21	−.06	.01	Polyamorous	−.04	−.13	.04	.01
Ideal-Warm	.41⁂	.33	.49	.01	Ideal-Warm	−.15⁑	−.25	−.06	.01
Ideal-Empowered	.13⁑	.05	.21	.11	Ideal-Empowered	.08	−.02	.17	.11
Ideal-Smart	.00	−.09	.08	.00	Ideal-Smart	.09*	.00	.17	.00
Ideal-Dependable	–	–	–	–	Ideal-Dependable	.03	−.06	.13	.00
Ideal-Aesthetic	.03	−.05	.10	.01	Ideal-Aesthetic	–	–	–	–
Ideal-Intimate	.18⁂	.10	.26	.00	Ideal-Intimate	.26⁂	.18	.35	.05
Ideal-Easy-going	.01	−.07	.09	.00	Ideal-Easy-going	−.03	−.11	.05	.00
Self-Mate-Value	−.02	−.10	.05	.00	Self-Mate-Value	.14⁂	.06	.22	.02
Ideal-Intimate (Adj. *R*^2^ = .26)					Ideal-Easy-going (Adj. *R*^2^ = .12)				
Female	−.06	−.14	.03	.00	Female	−.13⁑	−.22	−.04	.01
Binary-trans	−.04	−.12	.03	.00	Binary-trans	−.10*	−.18	−.02	.01
Non-binary	−.02	.02	.12	.00	Non-binary	.05	−.04	.14	.00
Same-attracted	.12⁑	−.05	.13	.01	Same-attracted	−.08	−.10	.01	.00
Multi-attracted	.07	−.02	.16	.00	Multi-attracted	−.06	−.15	.04	.00
Asexual	−.16⁂	−.24	−.09	.02	Asexual	−.01	−.09	.07	.00
Polyamorous	.12⁑	.04	.19	.01	Polyamorous	−.04	−.13	.04	.00
Ideal-Warm	.14⁑	.05	.24	.01	Ideal-Warm	.21⁂	.10	.31	.03
Ideal-Empowered	.08	−.01	.17	.00	Ideal-Empowered	.22⁂	.13	.32	.03
Ideal-Smart	.04	−.04	.12	.00	Ideal-Smart	−.02	−.10	.08	.00
Ideal-Dependable	.20⁂	.12	.29	.03	Ideal-Dependable	.01	−.08	.11	.00
Ideal-Aesthetic	.25⁂	.17	.33	.05	Ideal-Aesthetic	−.03	−.12	.06	.00
Ideal-Intimate	–	–	–	–	Ideal-Intimate	.08	−.01	.17	.00
Ideal-Easy-going	.07	−.01	.14	.00	Ideal-Easy-going	–	–	–	–
Self-Mate-Value	.05	−.03	.13	.00	Self-Mate-Value	−.01	−.09	.07	.00
Self-Warm (Adj. *R*^2^ = .34)					Self-Empowered (Adj. *R*^2^ = .37)				
Female	−.06	−.14	.02	.00	Female	−.10*	−.17	−.02	.01
Binary-trans	.01	−.06	.09	.00	Binary-trans	−.02	−.09	.05	.00
Non-binary	.03	−.05	.11	.00	Non-binary	.01	−.06	.08	.00
Same-attracted	.01	−.03	.05	.00	Same-attracted	.00	−.04	.05	.00
Multi-attracted	.02	−.07	.11	.00	Multi-attracted	−.02	−.11	.06	.00
Asexual	.05	−.02	.12	.00	Asexual	.00	−.08	.08	.00
Polyamorous	.01	−.06	.08	.00	Polyamorous	−.03	−.10	.04	.00
Ideal-Warm	.50⁂	.41	.59	.14	Ideal-Warm	−.01	−.09	.08	.00
Ideal-Empowered	−.06	−.14	.03	.00	Ideal-Empowered	.39⁂	.31	.47	.10
Ideal-Smart	.02	−.06	.10	.00	Ideal-Smart	−.01	−.08	.06	.00
Ideal-Dependable	.02	−.07	.11	.00	Ideal-Dependable	.06	−.03	.14	.00
Ideal-Aesthetic	−.13⁑	−.20	−.05	.01	Ideal-Aesthetic	.00	−.08	.09	.00
Ideal-Intimate	.13⁑	.05	.21	.01	Ideal-Intimate	.00	−.08	.07	.00
Ideal-Easy-going	.07	−.00	.14	.00	Ideal-Easy-going	.07	−.01	.14	.00
Self-Mate-Value	.12⁑	.05	.19	.01	Self-Mate-Value	.36⁂	.29	.43	.11

*p < .05, ⁑ *p* < .01, ⁂ *p* < .001. *sr*^2^ = semi-partial correlation. CI = confidence interval

Adj. *R*^2^ = significant adjusted *R*^2^

We observed that most identity and preference predictors significantly and positively predicted a preference for warm partners (*β* = 0.07 to 0.37). A higher preference for aesthetic partners was the only negative significant predictor, perhaps indicating that warm traits are less valued by those focused on physical appearance. A noticeable trend was self-perceived mate value predicting self-ratings on each trait dimension, which aligns with the literature (Csajbók & Berkics, 2017). Dependable self-ratings did not follow this trend, perhaps indicating this construct is inconsistently part of individuals’ construction of their mate value self-perceptions. The only significant gender or sexual identity predictor for self-ratings was assigned sex, with female participants having lower empowered and smart self-ratings.

We found it notable that assigned sex was not a significant predictor of preference for empowered partners in regression, as previous research might suggest. Self-perceived mate value and preferences for smart, dependable, and easy-going were the significant predictors in this model; apart from preferences for easy-going partners, these predictors were positively correlated with assigned sex and therefore could suppress the predictive utility of assigned sex. We performed relative importance analysis (Tonidandel et al., 2009) and dominance analysis (Lorenzo-Seva et al., 2010) to better understand how each predictor accounts for unique variance without suppression effects (Karpen, 2017). The results of these analyses conflicted and were thus inconclusive, so we instead considered the possibility that self-perceived mate value and preferences for smart and dependable partners mediated the relationship between assigned sex and preference for empowered partners. Our analyses supported this possibility for each mediator; we tested all three mediators in the same model and found that all direct and indirect effects were simultaneously significant within the same model, indicating a multiple mediation effect (Collier, 2020). These findings are reported in Table 7 and suggest that while the association between assigned sex and preference for empowered partners is valid, other common factors such as self-perceived mate value and preference patterns may influence that association. However, the veracity of this explanation requires further replication and validation.

**Table 7:** Results of analysis exploring multiple mediation of relationship between assigned sex and preference for empowered partners

Pathway	Direct Effect (*p*)	Indirect Effect (*p*)	Indirect Effect 95% CI	
			Lower Limit	Upper Limit
Assigned sex → Self-perceived mate value → Empowered trait preference	.36 (.002)	.05 (.001)	.02	.09
Assigned sex → Smart trait preference → Empowered trait preference	.23 (.011)	.14 (.002)	.07	.22
Assigned sex → Dependable trait preference → Empowered trait preference	.15 (.004)	.08 (.003)	.02	.21

Analyses were bootstrapped for 1000 sample estimates

### Multiple Regression Analyses Predicting Self-Perceived Mate Value Across Gender and Sexual Identity

In Table 8, we present a selection of multiple regression analyses predicting self-perceived mate value that are pertinent to the discussion in this paper and highlight broad trends in our findings. This decision was made to focus our reporting and discussion; a complete set of these analyses is available in Appendix B. Alternative models that explored the predictive utility of age, a combination of binary-trans and non-binary predictors, and combined majority and minority identity predictors did not significantly or substantially impact any analyses. We note a significant increase in self-perceived mate value in the bisexual subsample for self-ratings on the intimate (*β* = 0.22, *p* = .010) and aesthetic dimensions (*β* = 0.22, *p* = .010). For the pansexual subsample, we observed a non-significant decrease in self-perceived mate value for self-ratings on the intimate dimension (*β* = −0.10, *p* = .550), while aesthetic self-ratings were also non-significant predictors (*β* = 0.23, *p* = .123). Based on these results and others reported in this study, it appears bisexual participants in this sample had a consistent preference and self-perception of intimate and aesthetic traits that supersedes other factors of identity, preference, and self-perception.

**Table 8:** Selected multiple regression analysis results predicting self-perceived mate value with gender and sexual identity, and partner trait self-ratings (*N* = 549)

	*β*	*β* 95% CI				*β*	*β* 95% CI		
		Low	High	*sr*^2^			Low	High	*sr*^2^
Overall (*N* = 549, Adj. *R*^2^ = .43)					Multi-attracted (*n* = 167, Adj. *R*^2^ = .43)				
Female	.17⁂	.10	.24	.02	Female	.16*	.02	.29	.02
Binary-trans	−.04	−.10	.03	.00	Binary-trans	.00	−.11	.12	.00
Non-binary	−.05	−.12	.02	.00	Non-binary	−.06	−.18	.06	.00
Same-attracted	.03	−.04	.10	.00	Same-attracted	–	–	–	–
Multi-attracted	−.05	−.13	.02	.00	Multi-attracted	–	–	–	–
Asexual	−.12⁂	−.19	−.05	.01	Asexual	−.18⁑	−.31	−.05	.03
Polyamorous	.08*	.01	.14	.01	Polyamorous	.12	−.00	.24	.01
Self-Warm	.06	−.02	.14	.00	Self-Warm	.08	−.06	.22	.00
Self-Empowered	.26⁂	.18	.33	.04	Self-Empowered	.21⁑	.06	.36	.03
Self-Smart	−.01	−.07	.06	.00	Self-Smart	.00	−.23	.23	.00
Self-Dependable	−.06	−.14	.01	.00	Self-Dependable	−.06	−.19	.08	.00
Self-Aesthetic	.43⁂	.36	.50	.15	Self-Aesthetic	.41⁂	.28	.55	.13
Self-Intimate	.07	.00	.15	.00	Self-Intimate	.15*	.01	.28	.02
Self-Easy-going	.02	−.05	.09	.00	Self-Easy-going	−.04	−.17	.10	.00
Majority (*n* = 261, Adj. *R*^2^ = .44)					Minority (*n* = 288, Adj. *R*^2^ = .42)				
Female	.22⁂	.12	.32	.04	Female	.13*	.02	.23	.01
Binary-trans	–	–	–	–	Binary-trans	−.04	−.13	.05	.00
Non-binary	–	–	–	–	Non-binary	−.07	−.16	.03	.00
Same-attracted	–	–	–	–	Same-attracted	.06	−.14	.25	.00
Multi-attracted	–	–	–	–	Multi-attracted	−.02	−.21	.18	.00
Asexual	–	–	–	–	Asexual	−.13*	−.24	−.03	.01
Polyamorous	–	–	–	–	Polyamorous	.09	−.01	.18	.01
Self-Warm	−.03	−.15	.10	.00	Self-Warm	.12*	.00	.23	.01
Self-Empowered	.29⁂	.18	.41	.06	Self-Empowered	.24⁂	.13	.35	.04
Self-Smart	−.05	−.15	.05	.00	Self-Smart	.04	−.06	.14	.00
Self-Dependable	.03	−.08	.14	.00	Self-Dependable	−.13*	−.23	−.03	.01
Self-Aesthetic	.45⁂	.35	.55	.17	Self-Aesthetic	.42⁂	.32	.52	.13
Self-Intimate	.04	−.07	.16	.00	Self-Intimate	.09	−.01	.20	.01
Self-Easy-going	.09	−.01	.19	.01	Self-Easy-going	−.03	−.12	.07	.00
Male (*n* = 225, Adj. *R*^2^ = .43)					Female (*n* = 324, Adj. *R*^2^ = .43)				
Female	–	–	–	–	Female	–	–	–	–
Binary-trans	−.06	−.18	.05	.00	Binary-trans	−.03	−.12	.05	.00
Non-binary^a^	−.12*	−.23	−.01	.01	Non-binary	−.02	−.12	.07	.00
Same-attracted	.06	−.05	.17	.00	Same-attracted	−.02	−.11	.08	.00
Multi-attracted	.01	−.11	.12	.00	Multi-attracted	−.08	−.18	.01	.01
Asexual	−.10	−.20	.01	.01	Asexual	−.15⁂	−.24	−.06	.02
Polyamorous	.09	−.02	.20	.01	Polyamorous	.08	−.01	.17	.01
Self-Warm	.06	−.07	.19	.00	Self-Warm	.07	−.04	.18	.00
Self-Empowered	.34⁂	.22	.46	.08	Self-Empowered	.20⁂	10	.30	.03
Self-Smart	−.03	−.14	.08	.00	Self-Smart	.00	−.09	.09	.00
Self-Dependable	.01	−.11	.13	.00	Self-Dependable	−.11*	−.21	−.01	.01
Self-Aesthetic	.47⁂	.35	.58	.16	Self-Aesthetic	.42⁂	.33	.51	.14
Self-Intimate	−.06	−.18	.07	.00	Self-Intimate	.16⁑	.06	.26	.02
Self-Easy-going	.04	−.07	.15	.00	Self-Easy-going	.02	−.08	.11	.00
Man (*n* = 217, Adj. *R*^2^ = .45)					Woman (*n* = 288, Adj. *R*^2^ = .43)				
Female	–	–	–	–	Female	–	–	–	–
Binary-trans	.04	−.07	.15	.00	Binary-trans	−.09*	−.18	−.00	.01
Non-binary	–	–	–	–	Non-binary	–	–	–	–
Same-attracted	.09	−.01	.20	.01	Same-attracted	−.05	−.15	.04	.00
Multi-attracted	.02	−.09	.12	.00	Multi-attracted	−.07	−.17	.03	.00
Asexual	−.13*	−.23	−.02	.01	Asexual	−.13⁑	−.23	−.04	.02
Polyamorous	−.01	−.11	.09	.00	Polyamorous	.01	−.08	.10	.00
Self-Warm	.02	−.11	.16	.00	Self-Warm	.08	−.03	.19	.00
Self-Empowered	.29⁂	.17	.40	.06	Self-Empowered	.23⁂	.12	.34	.03
Self-Smart	.01	−.10	.12	.00	Self-Smart	−.02	−.12	.07	.00
Self-Dependable	.02	−.10	.14	.00	Self-Dependable	−.11*	−.21	−.01	.01
Self-Aesthetic	.50⁂	.38	.61	.18	Self-Aesthetic	.41⁂	.31	.51	.13
Self-Intimate	−.01	−.14	.13	.00	Self-Intimate	.12*	.02	.23	.01
Self-Easy-going	.04	−.07	.15	.00	Self-Easy-going	.06	−.03	.16	.00

*p < .05, ⁑ *p* < .01, ⁂ *p* < .001. *sr*^2^ = semi-partial correlation. CI = confidence interval. Adj. *R*^2^ = adjusted *R*^2^

^a^ interpret this predictor with caution due to low subsample sizes (male non-binary participant *n* = 7)

An interesting finding that we highlight here is that warm self-ratings did not significantly predict self-perceived mate value in any subsample except for the combined minority identity subsample. This could be due to underpowered specific minority identity subsamples, but it is still curious, considering how warm trait preferences were linked with identity and preference predictors. However, self-perceived mate value did not predict preference for warm traits. We can only speculate as to why warm traits may not be associated with self-perceived mate value; it may be that warm traits do not overcome traits that individuals consider more relevant to their self-perceived mate value, such as aesthetic and empowered traits. Indeed, these traits strongly, consistently, and significantly predicted higher self-perceived mate value in these analyses (aesthetic, *β* = 0.32 to 0.50, empowered, *β* = 0.20 to 0.34). Other consistent significant predictors observed were being assigned sex (*β* = 0.16 to 0.19) and asexual identification (*β* = −0.09 to −0.15).

### Summary of Findings

We found that trait preference dimensions in an Australian sample were similar to those observed in previous studies (Csajbók & Berkics, 2017; Fletcher et al., 1999) and that these dimensions were appropriate in majority and minority subsamples. When comparing assigned sex and sexual orientation identity groups, we found little difference in romantic partner selection experiences, but significant differences on beliefs, trait preferences, and self-perception. Gender and sexual identity seemed to interact in some areas of romantic partner selection, but these effects were small. Our regression analyses further revealed how the intersection of gender and sexual identity may influence romantic partner selection, especially from the perspective of majority and minority populations. These regression results also indicated that female participants’ preference for empowered partners may be better explained by their self-perceptions and other preferences; this finding highlights mediation effects as a potential explanation for conflicting findings around sex differences in trait preferences.

## Discussion

The findings of our study indicate that sexual identity should be considered alongside assigned sex and/or gender identity when investigating partner selection processes. Sexual orientation identities were significantly different on 10 of 23 test variables in factorial analyses alongside assigned sex as a group variable. Significant differences on empowered and aesthetic self-ratings, and challenges finding a romantic partner were unique to sexual orientation identity, indicating potentially distinct influences on self-perception and experience. Our findings also support the idea of a complex relationship between identity and partner selection experiences through our observation of six significant interaction effects between assigned sex and sexual orientation identity groups and intersectional factors in regression analyses. It is important to note that we observed many non-significant differences and predictors in our analyses that indicate assigned sex and sexual identity groups may be similar in more ways than they differ. Overall, these findings highlight the interplay of assigned sex and sexual identity in partner selection processes, add nuance to previous findings regarding assigned sex differences, and suggest that both differences and similarities between minority and majority groups are important to consider if we are to understand individuals’ complex partner selection experiences.

Our comparative analyses replicated other researchers’ findings on assigned sex differences, wherein males prefer aesthetic partners and females prefer empowered partners (Buss et al., 2020; Eastwick & Finkel, 2008; March & Bramwell, 2012), but we did not observe the difference in preference for empowered partners in our regression analyses. Instead, self-perceived mate value and higher preferences for smart, dependable, and easy-going partners appeared to be associated with a higher preference for empowered partners. Further analysis revealed that these predictors, apart from a preference for easy-going partners, mediated the association between assigned sex and preference for empowered partners. The regression and mediation results indicate that this constellation of preferences and self-perception may influence individuals’ preference for empowered partners in interaction with, or beyond, the influence of assigned sex. These findings could indicate dating market forces (Wood & Brumbaugh, 2009) where higher mate value permits a broad pattern of higher preferences (Conroy-Beam et al., 2022)—perhaps centered on empowered traits. Alternatively, this finding could be representative of partner evaluation processes (Conroy-Beam et al., 2022) that center on empowered traits and exert influence across assigned sex or changing gender role norms that may lead males to appreciate empowered traits in females and reduce the strength of assigned sex associations (Eastwick et al., 2024; Gale et al., 2024), although there is also some evidence that these norms may be persistent (Underwood, 2023). Any of these alternatives challenge a consistently observed assigned sex difference but would require replication and testing of alternative explanations; it would also be interesting to see how qualitative investigations could explore the lived experience of preferring or not preferring empowered partners.

Our findings suggest that participants found it more challenging to find a partner if they had lower self-perceived mate value, which aligns with market theories of partner selection (Bredow et al., 2011). We observed further support for market theories of partner selection when participants with higher self-perceived mate value made quicker decisions about partner suitability, perhaps because they quickly eliminate prospective partners they perceive as possessing lower mate value (Conroy-Beam et al., 2022). Quick decisions on partner suitability became a less common experience with a higher preference for warm partners, perhaps because warm traits might be more challenging to discern quickly. However, some studies have found the opposite (South Palomares & Young, 2017). This finding frames the partner selection process as potentially longer or multi-phased for some people, which could result in conflicting levels of interest or commitment between prospective partners (Devenport et al., 2023; Eastwick et al., 2022; Günaydin et al., 2013; Levinger & Snoek, 1972). An interesting direction for future research could be to investigate how individual differences in partner preference or the partner selection process might impact the duration or timing of partner selection decisions (Conroy-Beam et al., 2022; Devenport et al., 2023).

Same-attracted participants found it significantly more challenging to find partners than different-attracted participants. This greater challenge faced by same-attracted participants may be due to having a smaller pool of prospective partners to select from (Wilson et al., 2020). Multi-attracted participants’ challenges in finding a partner were not significantly different from different- or same-attracted participants, a finding supported in the regression analyses. A deeper investigation of this finding revealed that male multi-attracted participants reported similar levels of challenge to same-attracted participants. In contrast, female multi-attracted participants reported similar levels of challenge to different-attracted participants. One explanation for this discrepancy could be that while male multi-attracted individuals may benefit from a larger pool of prospective partners, they also experience elevated biphobia in comparison with female multi-attracted individuals (Ess et al., 2023; Gleason et al., 2019). We suggest that this elevated biphobia could make it harder to find accepting partners and offset the benefits of their larger prospective partner pool. This pattern of findings highlights how minority groups can have disadvantages arising from their minority nature, such as a lower pool of prospective partners, but also face disadvantages from minority stress and stigma, which can have separate or compounding effects.

We observed a trend that may indicate the challenges that norms present to those identifying with a minority identity, wherein the different-attracted and multi-attracted groups would each yield either the highest or lowest scores with the same-attracted group somewhere between. We suggest that multi-attracted participants challenge majority norms in at least two ways: not being exclusively heterosexual and having multiple suitable partner types based on assigned sex and/or gender identity. This dual challenge may contribute to a lower preference for traits that the majority samples consistently and strongly prefer, such as aesthetic and empowered traits (Eastwick et al., 2014; Thomas et al., 2020). Our regression analyses support this possibility when considering preference for aesthetic partners but not when predicting preference for empowered partners. Further research might investigate if multi-attracted individuals perceive norms to be influencing their preferences or if there are alternative explanations; future studies should unpack these ideas further.

We note that preference for warm traits was central in the partner selection process as five of the six preferences and five of the six identity variables were associated with a preference for warm partners. Female participants strongly preferred warm partners, an inconsistent observation in the literature (Fletcher et al., 1999, 2004). Furthermore, identifying with a minority gender or sexual identity, apart from non-binary gender, predicted a higher preference for warm partners, corresponding with majority identification predicting a lower preference for warm partners. As discussed, romantic experiences are potentially difficult for individuals of minority identities (Blair & Hoskin, 2019; Friedman et al., 2014; Vares, 2018). We suggest that these difficulties may logically lead to a higher preference for a considerate, compassionate, supportive, open-minded, and patient partner for individuals of minority identity. Lastly, we note that preferences for warm partners and other traits were generally high, which is likely best interpreted as the group with greater preference placing high importance on those traits, as opposed to the other groups placing less importance on them. Many of these group differences were small, possibly due to the normative desirability of many traits. It may mean that these differences do not manifest or impact each individual’s experience. However, there remains an indication that gender and sexual identity generally influence preference and, therefore, experience.

Participants’ trait preferences were strongly associated with their self-ratings on the corresponding trait, highlighting the importance of perceiving partners as similar (Montoya et al., 2008). Participants’ preference for more aesthetic partners was associated with their preference for more intimate partners, which in turn was associated with a preference for aesthetic partners, a finding which we argue could be due to increased sexual desire toward more aesthetic partners (Hahn et al., 2016; Hughes et al., 2010). A higher preference for intimate partners was predicted by identifying as polyamorous, aligning with this population’s general desire for more emotional intimacy and perhaps sexual intimacy (Morrison et al., 2013). A lower preference for intimate partners was predicted by identifying as asexual, perhaps due to the implied sexual attraction of the intimate dimension traits. Conversely, we found that sexual attraction identity did not predict a preference for aesthetic partners, highlighting the distinction between aesthetic and sexual attraction that asexual populations report (Scheller et al., 2023).

Across our subsample regression analyses, identifying as asexual consistently predicted lower self-perceived mate value. Asexuality challenges strong societal norms around sexual attraction, potentially resulting in self-perceptions of non-normality (Maxwell, 2017; Vares, 2018). These self-perceptions may connect to this particularly resilient trend of lower self-perceived mate value and warrant further attention in future research. However, we know that small subsample sizes may have resulted in lower predictive utility for minority gender and sexual identities in our regression analyses, with the risk of some predictive relationships remaining undetected. Multi-attracted participants also had significantly lowered self-perceived mate value compared to different- and same-attracted groups. However, multi-attracted identity did not significantly account for variance in self-perceived mate value in the broader regression model. This finding and others require replication, but there is still an indication that identity-based experiences influence self-perceived mate value for some populations. In contrast, the contribution of other populations’ identities may not exceed other self-perceptions, such as empowered and aesthetic self-ratings.

We observed that female participants reported consistently higher self-perceived mate value, a finding not usually observed in previous research (Edlund & Sagarin, 2014; Gillen et al., 2016). One explanation for our finding is the sexualization of women, which previous research has linked to positive self-image through self-objectification (Smolak & Murnen, 2011). However, links between sexualization and body dissatisfaction raise a challenge to this idea (Ward, 2016), especially given our findings that suggest aesthetic ratings were strongly and consistently associated with many of the variables in our study. A further complication in this narrative is that being a trans woman predicted lower self-perceived mate value in our study, but being a trans man did not. Trans men’s birth-assigned sex (female) may be responsible for this finding. Trans men are reportedly less appealing as prospective partners for majority populations, a trend that may be due to culturally hegemonic ‘femmephobia’ or privileging of masculine gender expressions (Blair & Hoskin, 2019; Pollitt et al., 2023). We believe these findings and related considerations provide rich directions for future research, from exploring group differences or similarities in self-perceived mate value to investigating links with lived transgender experience.

In our study, self-perceived mate value was strongly predicted by empowered and aesthetic self-ratings, consistent with the centrality of these traits observed in the literature (Thomas et al., 2020; Williams & Sulikowski, 2020). We note that Csajbók and Berkics (2017) also observed this predictive utility, although their status and dominance dimensions are somewhat combined within the empowered dimension in the current study. We note that Csajbók and Berkics did not measure dependable traits in their study, but in our study, these self-ratings displayed predictive utility in minority and female subsamples. Gender and sexual identity did not account for variance in self-perceived mate value beyond what previous research has observed but did significantly account for unique variance, unlike those previous observations (Csajbók & Berkics, 2017; Csajbók et al., 2019). This could indicate a unique contribution of identity to self-perceived mate value that could influence individuals’ partner selection experiences that future research should not ignore.

We are unaware of any studies investigating experience, trait preference, and/or mate value that also include and integrate the diverse range of gender and sexual identities that were features of this study. Minority populations are proportionally smaller than majority populations; therefore, obtaining a sufficiently powered sample or subsample can be difficult. This was certainly the case in our study, despite the targeted recruitment of participants identifying with minority gender and sexual identities. Unfortunately, it was beyond the practical scope of the study to recruit further than reported. While the underpowered nature of some analyses prompts caution in interpreting results, we believe that the findings have value in furthering our understanding of how gender and sexual identity contribute to partner selection processes.

Our findings suggest that a larger set of trait preference dimensions may be viable across and within majority and minority gender and sexual identity, at least within a contemporary Australian sample. The traits measured in this study were not as extensive as those in Fletcher et al. (1999), and our factor analysis is not as robust as Csajbók and Berkics (2017). However, the dimensions observed here broadly match those established in earlier research. Key informants of diverse gender and/or sexual identities who reviewed the traits for suitability before the study improved the validity of traits used across different identities. The consistency we observed in our dimensional structure across majority and minority subsamples is important, but confidence in this consistency requires more rigorous testing and replication. We suggest that future research could investigate how to better express and measure items that capture specific partner traits, perhaps distinguishing these from partnered traits (e.g., sexually compatible) and capturing easy-going traits such as a sense of humor. We also recommend a more thorough investigation of trait preference dimensions and attempts to unify various measures in this area.

Our efforts to capture intersectional factors quantitatively were partially successful, with intersections between gender and sexual orientation identities captured in our comparative analyses, and intersections within sexual identity in our regression analyses. However, the smaller subsample sizes of many identity groups prevented more complex analyses and necessitated our collapsing of all minority identity classification into a group for analysis. Despite these issues, our findings show there is potential in investigating intersectional factors quantitatively, and we recommend that future research further explores intersections between gender, sexual, and other identities (e.g., ethnicity and sexual identity, Galupo et al., 2019). We also note that the influence of other individual differences, such as openness (Allen & Robson, 2020), on partner selection experiences remains an underexplored area of research. Future research might explore other individual differences that identity sometimes forms around, such as neurodivergence and disability (Buijsman et al., 2023; Mogavero & Hsu, 2020; Saltes, 2013), especially in interaction with gender and/or sexual identity (Lewis et al., 2021). It is also important to note that we did not include a measure of social desirability bias in this study, which may account for some of the variation in responses observed (Krumpal, 2013). While many partner selection studies rely on anonymity to mitigate social desirability bias, future research would benefit from considering the impacts of such biases more formally.

In conclusion, our study provides preliminary evidence that gender and sexual identities have differential, concurrent, and interactive impacts on partner selection experiences, beliefs, preferences, and self-perceptions. Social norms for romantic and sexual relationships emerged as a potential explanation for the pattern of differences and relationships across minority gender and sexual identities. It is important to reiterate that there were also instances where we observed no impact of gender or sexual identity that may indicate similarities between assigned sex and majority–minority groups. We observed intersectional factors, further illustrating the complexity inherent in partner selection processes. Our findings highlight this complexity, contribute to knowledge of partner selection, and enable future research that further explores how people select romantic partners.

## Supplementary Information

Below is the link to the electronic supplementary material.

Supplementary file1 (PDF 431 kb)

## Data Availability

The data that support the findings of this study are not publicly available due to ethical restrictions.

## Footnotes

1 Cisgender refers to individuals whose gender identity matches their assigned sex at birth (assigned sex in this paper). Heterosexual refers to individuals who are exclusively attracted to partners of one assigned sex or gender other than their own. We use the terms majority and minority to describe the statistical proportion groups throughout this paper.

## Code Availability

There are no applicable applications or code associated with this study.

## References

[CR1] Albury, K., Dietzel, C., Pym, T., Vivienne, S., & Cook, T. (2021). Not your unicorn: Trans dating app users’ negotiations of personal safety and sexual health. *Health Sociology Review,**30*(1), 72–86. 10.1080/14461242.2020.1851610 33622202 10.1080/14461242.2020.1851610

[CR2] Arístegui, I., Castro Solano, A., & Buunk, A. P. (2018). Mate preferences in Argentinean transgender people: An evolutionary perspective. *Personal Relationships,**25*(3), 330–350. 10.1111/pere.12247

[CR3] Arístegui, I., Castro Solano, A., & Buunk, A. P. (2019). Do transgender people respond according to their biological sex or their gender identity when confronted with romantic rivals? *Evolutionary Psychology,**17*(2). 10.1177/1474704919851139 31109194 10.1177/1474704919851139 PMC10480994

[CR4] Birkás, B., Láng, A., & Meskó, N. (2018). Self-rated attractiveness moderates the relationship between dark personality traits and romantic ideals in women. *Psychological Reports,**121*(1), 184–200. 10.1177/0033294117738021 29298572 10.1177/0033294117738021

[CR5] Blair, K. L., & Hoskin, R. A. (2019). Transgender exclusion from the world of dating: Patterns of acceptance and rejection of hypothetical trans dating partners as a function of sexual and gender identity. *Journal of Social and Personal Relationships,**36*(7), 2074–2095. 10.1177/0265407518779139

[CR6] Bollen, K. A. (1989). *Structural equations with latent variables*. John Wiley and Sons.

[CR7] Bostwick, W., & Hequembourg, A. (2014). ‘Just a little hint’: Bisexual-specific microaggressions and their connection to epistemic injustices. *Culture, Health and Sexuality,**16*(5), 488–503. 10.1080/13691058.2014.889754 24666221 10.1080/13691058.2014.889754

[CR8] Boxer, C., Noonan, M., & Whelan, C. (2013). Measuring mate preferences: A replication and extension. *Journal of Family Issues,**36*(2), 163–187. 10.1177/0192513x13490404

[CR9] Bredow, C. A., Huston, T. L., & Glenn, N. D. (2011). Market value, quality of the pool of potential mates, and singles’ confidence about marrying. *Personal Relationships,**18*(1), 39–57. 10.1111/j.1475-6811.2010.01302.x

[CR10] Brown, M., Brown, M. R., & Buckner, Z. (2023). Whither the silly goose: Clarifying women’s preference for men’s successful humor displays across mating contexts and social affordance judgments. *Evolutionary Behavioral Sciences.*10.1037/ebs0000338

[CR11] Buijsman, R., Begeer, S., & Scheeren, A. M. (2023). ‘Autistic person’ or ‘person with autism’? Person-first language preference in Dutch adults with autism and parents. *Autism,**27*(3), 788–795. 10.1177/13623613221117914 35957517 10.1177/13623613221117914 PMC10074744

[CR12] Buss, D. M. (1989). Sex differences in human mate preferences: Evolutionary hypotheses tested in 37 cultures. *Behavioral and Brain Sciences,**12*, 1–14. 10.1017/s0140525x00023992

[CR13] Buss, D. M., Durkee, P. K., Shackelford, T. K., Bowdle, B. F., Schmitt, D. P., Brase, G. L., Choe, J. C., & Trofimova, I. (2020). Human status criteria: Sex differences and similarities across 14 nations. *Journal of Personality and Social Psychology,**119*(5), 979–998. 10.1037/pspa0000206 32463270 10.1037/pspa0000206

[CR14] Buss, D. M., & Schmitt, D. P. (2019). Mate preferences and their behavioral manifestations. *Annual Review of Psychology,**70*, 77–110. 10.1146/annurev-psych-010418-103408 30230999 10.1146/annurev-psych-010418-103408

[CR15] Campbell, L., & Fletcher, G. (2015). Romantic relationships, ideal standards, and mate selection. *Current Opinion in Psychology,**1*, 97–100. 10.1016/j.copsyc.2015.01.007

[CR16] Carels, R. A., Miller, J. C., Shonrock, A. T., Byrd, R., Sall, K. E., & Carraway, M. (2022). Weight stigma among heterosexual and sexual minority individuals: Dating and hiring preferences. *Stigma and Health,**7*(4), 481–490. 10.1037/sah0000382

[CR17] Cole, E. R. (2009). Intersectionality and research in psychology. *American Psychologist,**64*(3), 170–180. 10.1037/a0014564 19348518 10.1037/a0014564

[CR93] Collier, J. (2020).* Applied structural equation modeling using AMOS: Basic to advanced techniques*. Routledge

[CR18] Conley, T. D., Moors, A. C., Matsick, J. L., Ziegler, A., & Valentine, B. A. (2011). Women, men, and the bedroom: Methodological and conceptual insights that narrow, reframe, and eliminate gender differences in sexuality. *Current Directions in Psychological Science,**20*(5), 296–300. 10.1177/0963721411418467

[CR19] Conroy-Beam, D., Walter, K. V., & Duarte, K. (2022). What is a mate preference? Probing the computational format of mate preferences using couple simulation. *Evolution and Human Behavior,**43*(6), 510–526. 10.1016/j.evolhumbehav.2022.09.002

[CR20] Crenshaw, K. W. (2017). *On intersectionality: Essential writings*. The New Press. https://scholarship.law.columbia.edu/books/255/

[CR21] Csajbók, Z., & Berkics, M. (2017). Factor, factor, on the whole, who’s the best fitting of all? *Personality and Individual Differences,**114*, 92–102. 10.1016/j.paid.2017.03.044

[CR22] Csajbók, Z., Havlíček, J., Demetrovics, Z., & Berkics, M. (2019). Self-perceived mate value is poorly predicted by demographic variables. *Evolutionary Psychology,**17*(1). 10.1177/1474704919829037 30816069 10.1177/1474704919829037 PMC10481051

[CR23] Devenport, S., Davis-McCabe, C., & Winter, S. (2023). A critical review of the literature regarding the selection of long-term romantic partners. *Archives of Sexual Behavior,**52*(7), 3025–3042. 10.1007/s10508-023-02646-y 37420089 10.1007/s10508-023-02646-y PMC10684645

[CR24] Eastwick, P. W., & Finkel, E. J. (2008). Sex differences in mate preferences revisited: Do people know what they initially desire in a romantic partner? *Journal of Personality and Social Psychology,**94*(2), 245–264. 10.1037/0022-3514.94.2.245 18211175 10.1037/0022-3514.94.2.245

[CR25] Eastwick, P. W., Finkel, E. J., & Joel, S. (2023). Mate evaluation theory. *Psychological Review,**130*(1), 211–241. 10.1037/rev0000360 35389716 10.1037/rev0000360

[CR26] Eastwick, P. W., Joel, S., Carswell, K. L., Molden, D. C., Finkel, E. J., & Blozis, S. A. (2022). Predicting romantic interest during early relationship development: A preregistered investigation using machine learning. *European Journal of Personality, 37,* 276–312. 10.1177/08902070221085877

[CR27] Eastwick, P. W., Sparks, J., Finkel, E. J., Meza, E. M., Adamkovič, M., Adu, P., Ai, T., Akintola, A. A., Al-Shawaf, L., Apriliawati, D., Arriaga, P., Aubert-Teillaud, B., Baník, G., Barzykowski, K., Batres, C., Baucom, K. J., Beaulieu, E. Z., Behnke, M., Butcher, N., … Coles, N. A. (2025). A worldwide test of the predictive validity of ideal partner preference matching. *Journal of Personality and Social Psychology, 128*, 123–146. 10.1037/pspp0000524 10.1037/pspp0000524 PMC12622239 39480282

[CR28] Eastwick, P. W., Luchies, L. B., Finkel, E. J., & Hunt, L. L. (2014). The predictive validity of ideal partner preferences: A review and meta-analysis. *Psychological Bulletin,**140*(3), 623–665. 10.1037/a0032432 23586697 10.1037/a0032432

[CR29] Edlund, J., & Sagarin, B. (2010). Mate value and mate preferences: An investigation into decisions made with and without constraints. *Personality and Individual Differences,**49*(8), 835–839. 10.1016/j.paid.2010.07.004

[CR30] Edlund, J., & Sagarin, B. (2014). The Mate Value Scale. *Personality and Individual Differences,**64*, 72–77. 10.1016/j.paid.2014.02.005

[CR31] Ess, M., Burke, S. E., & LaFrance, M. (2023). Gendered anti-bisexual bias: Heterosexual, bisexual, and gay/lesbian people’s willingness to date sexual orientation ingroup and outgroup members. *Journal of Homosexuality,**70*(8), 1461–1478. 10.1080/00918369.2022.2030618 35112988 10.1080/00918369.2022.2030618

[CR32] Faul, F., Erdfelder, E., Buchner, A., & Lang, A.-G. (2009). Statistical power analyses using G*Power 3.1: Tests for correlation and regression analyses. *Behavior Research Methods,**41*, 1149–1160. 10.3758/BRM.41.4.1149 19897823 10.3758/BRM.41.4.1149

[CR33] Forde, A. (2011). Evolutionary theory of mate selection and partners of trans people: A qualitative study using interpretative phenomenological analysis. *The Qualitative Report,**16*, 1407–1434. 10.46743/2160-3715/2011.1306

[CR34] Fletcher, G., Simpson, J., Thomas, G., & Giles, L. (1999). Ideals in intimate relationships. *Journal of Personality and Social Psychology,**76*, 72–89. 10.1037/0022-3514.76.1.72 9972554 10.1037//0022-3514.76.1.72

[CR35] Fletcher, G. J., Tither, J. M., O’Loughlin, C., Friesen, M., & Overall, N. (2004). Warm and homely or cold and beautiful? Sex differences in trading off traits in mate selection. *Personality and Social Psychology Bulletin,**30*(6), 659–672. 10.1177/0146167203262847 15155031 10.1177/0146167203262847

[CR36] Friedman, M. R., Dodge, B., Schick, V., Herbenick, D., Hubach, R. D., Bowling, J., Goncalves, G., Krier, S., & Reece, M. (2014). From bias to bisexual health disparities: Attitudes toward bisexual men and women in the United States. *LGBT Health,**1*(4), 309–318. 10.1089/lgbt.2014.0005 25568885 10.1089/lgbt.2014.0005 PMC4283842

[CR37] Gale, M., Torbay, R., & Lykins, A. D. (2024). Visual attention to evolutionarily relevant information by heterosexual men and women while viewing mock online dating profiles. *Archives of Sexual Behavior,**53*(8), 3073–3085. 10.1007/s10508-024-02950-1 39009742 10.1007/s10508-024-02950-1 PMC11335984

[CR38] Galupo, M. P., Taylor, S. M., & Cole, D., Jr. (2019). “I am double the bi”: Positive aspects of being both bisexual and biracial. *Journal of Bisexuality,**19*(2), 152–168. 10.1080/15299716.2019.1619066

[CR39] García-Pérez, M. A. (2023). Use and misuse of corrections for multiple testing. *Methods in Psychology,**8*, Article 100120. 10.1016/j.metip.2023.100120

[CR40] Gillen, M., Collisson, B., Murtagh, M., Browne, B., & McCutcheon, L. (2016). Additional psychometric data for the Mate Value Scale. *Journal of Relationships Research,**7*, Article e7. 10.1017/jrr.2016.7

[CR41] Gleason, N., Vencill, J. A., & Sprankle, E. (2019). Swipe left on the bi guys: Examining attitudes toward dating and being sexual with bisexual individuals. *Journal of Bisexuality,**18*(4), 516–534. 10.1080/15299716.2018.1563935

[CR42] Griffiths, D. A., & Armstrong, H. L. (2023). “They were talking to an idea they had about me”: A qualitative analysis of transgender individuals’ experiences using dating apps. *Journal of Sex Research,**61*(1), 119–132. 10.1080/00224499.2023.2176422 36799719 10.1080/00224499.2023.2176422

[CR43] Günaydin, G., Selcuk, E., & Hazan, C. (2013). Finding the one: A process model of human mate selection. In C. Hazan & M. I. Campa (Eds.), *Human bonding: The science of affectional ties* (pp. 103–131). The Guilford Press.

[CR44] Hahn, A. C., Fisher, C. I., DeBruine, L. M., & Jones, B. C. (2016). Sex-specificity in the reward value of facial attractiveness. *Archives of Sexual Behavior,**45*, 871–875. 10.1007/s10508-015-0509-1 25868402 10.1007/s10508-015-0509-1

[CR45] Hall, J. A. (2015). Sexual selection and humor in courtship: A case for warmth and extroversion. *Evolutionary Psychology, 13*. 10.1177/1474704915598918 37924189 10.1177/1474704915598918 PMC10426839

[CR46] Hall, W. J., Dawes, H. C., & Plocek, N. (2021). Sexual orientation identity development milestones among lesbian, gay, bisexual, and queer people: A systematic review and meta-analysis. *Frontiers in Psychology,**12*, Article 753954. 10.3389/fpsyg.2021.753954 34777153 10.3389/fpsyg.2021.753954 PMC8581765

[CR47] Hill, R. (1945). Campus values in mate selection. *Journal of Home Economics,**37*(9), 554–558.

[CR48] Hughes, S. M., Farley, S. D., & Rhodes, B. C. (2010). Vocal and physiological changes in response to the physical attractiveness of conversational partners. *Journal of Nonverbal Behavior,**34*, 155–167. 10.1007/s10919-010-0087-9

[CR49] Hyde, J. S., Bigler, R. S., Joel, D., Tate, C. C., & van Anders, S. M. (2019). The future of sex and gender in psychology: Five challenges to the gender binary. *American Psychologist,**74*(2), 171. 10.1037/amp0000307 30024214 10.1037/amp0000307

[CR50] Ip, I. M. H., Honey, A., & McGrath, M. (2022). ‘Doing’ dating: A cross-sectional survey of young adults (18–35 years) in Australia and Hong Kong. *Australian Occupational Therapy Journal,**69*(3), 233–242. 10.1111/1440-1630.12785 35040135 10.1111/1440-1630.12785

[CR51] Kade, T. (2021). “Hey, by the way, I’m transgender”: Transgender disclosures as coming out stories in social contexts among trans men. *Socius,**7*. 10.1177/23780231211039389

[CR52] Karpen, S. C. (2017). Misuses of regression and ANCOVA in educational research. *American Journal of Pharmaceutical Education,**81*(8), 6501. 10.5688/ajpe6501 29200454 10.5688/ajpe6501 PMC5701329

[CR53] Krumpal, I. (2013). Determinants of social desirability bias in sensitive surveys: A literature review. *Quality and Quantity,**47*(4), 2025–2047. 10.1007/s11135-011-9640-9

[CR54] Levinger, G., & Snoek, J. (1972). *Attraction in relationship: A new look at interpersonal attraction*. General Learning Press.

[CR55] Lewis, L. F., Ward, C., Jarvis, N., & Cawley, E. (2021). “Straight sex is complicated enough!”: The lived experiences of autistics who are gay, lesbian, bisexual, asexual, or other sexual orientations. *Journal of Autism and Developmental Disorders,**51*, 2324–2337. 10.1007/s10803-020-04696-w 32968942 10.1007/s10803-020-04696-w

[CR56] Li, N. P., & Meltzer, A. L. (2015). The validity of sex-differentiated mate preferences: Reconciling the seemingly conflicting evidence. *Evolutionary Behavioral Sciences,**9*(2), 89–106. 10.1037/ebs0000036

[CR57] Li, N., Yong, J., Tov, W., Sng, O., Fletcher, G., Valentine, K., Jiang, Y., & Balliet, D. (2013). Mate preferences do predict attraction and choices in the early stages of mate selection. *Journal of Personality and Social Psychology,**105*(5), 757–776. 10.1037/a0033777 23915041 10.1037/a0033777

[CR58] Lorenzo-Seva, U., Ferrando, P. J., & Chico, E. (2010). Two SPSS programs for interpreting multiple regression results. *Behavior Research Methods,**42*(1), 29–35. 10.3758/BRM.42.1.29s 20160283 10.3758/BRM.42.1.29

[CR59] March, E., & Bramwell, A. (2012). Sex differences in mate preferences in Australia: Exploring evolutionary and social-economic theories. *Journal of Relationships Research,**3*, 18–23. 10.1017/jrr.2012.3

[CR60] Maxwell, D. (2017). *It’s not just about sex: Asexual identity and intimate relationship practices.* Doctoral dissertation, University of York. https://www.semanticscholar.org/paper/It’s-not-just-about-sex-%3A-asexual-identity-andMaxwell/10b156486b41e3ebc2b5efc4f460c80afb1ca94c

[CR61] Mitchell, K. M., & Knittel, M. L. (2023). Navigating the role of LGBTQ+ identity in self-disclosure and strategies used for uncertainty reduction in online dating. *Journal of Sex Research,**60*(5), 645–655. 10.1080/00224499.2023.2179009 36800920 10.1080/00224499.2023.2179009

[CR62] Mogavero, M. C., & Hsu, K. H. (2020). Dating and courtship behaviors among those with autism spectrum disorder. *Sexuality and Disability,**38*, 355–364. 10.1007/s11195-019-09565-8

[CR63] Montoya, R., Horton, R., & Kirchner, J. (2008). Is actual similarity necessary for attraction? A meta-analysis of actual and perceived similarity. *Journal of Social and Personal Relationships,**25*(6), 889–922. 10.1177/0265407508096700

[CR64] Moore, M. R. (2012). Intersectionality and the study of black, sexual minority women. *Gender and Society,**26*(1), 33–39. 10.1177/0891243211427031

[CR65] Morrison, T. G., Beaulieu, D., Brockman, M., & Beaglaoich, C. Ó. (2013). A comparison of polyamorous and monoamorous persons: Are there differences in indices of relationship well-being and sociosexuality? *Psychology and Sexuality,**4*(1), 75–91. 10.1080/19419899.2011.631571

[CR66] Obadia, J. (2020). Responsibility, respectability, recognition, and polyamory: Lessons in subject formation in the age of sexual identity. *Feminist Studies,**46*(2), 287–315. 10.1353/fem.2020.0040

[CR67] Park, J., & van Leeuwen, F. (2015). Evolutionary perspectives on social identity. In V. Zeigler-Hill, L. Welling, & T. Shackelford (Eds.), *Evolutionary perspectives on social psychology* (pp. 115–125). Springer. 10.1007/978-3-319-12697-5_9

[CR68] Pollitt, A. M., Blair, K. L., & Lannutti, P. J. (2023). A review of two decades of LGBTQ-inclusive research in JSPR and PR. *Personal Relationships,**30*(1), 144–173. 10.1111/pere.12432 PMC12735361 41451113

[CR69] Qian, Y. (2022). Disruption or reproduction? Nativity, gender and online dating in Canada. *Internet Research,**32*(4), 1264–1287. 10.1108/INTR-10-2020-0547

[CR70] Robinson, M. (2017). Using multi-item psychometric scales for research and practice in human resource management. *Human Resource Management,**2017*, 1–12. 10.1002/hrm.21852

[CR71] Rubin, M. (2021). When to adjust alpha during multiple testing: A consideration of disjunction, conjunction, and individual testing. *Synthese,**199*(3–4), 10969–11000. 10.1007/s11229-021-03276-4

[CR72] Saltes, N. (2013). Disability, identity and disclosure in the online dating environment. *Disability and Society,**28*(1), 96–109. 10.1080/09687599.2012.695577

[CR73] Scheller, M., de Sousa, A. A., Brotto, L. A., & Little, A. C. (2023). The role of sexual and romantic attraction in human mate preferences. *Journal of Sex Research,**61*(2), 299–312. 10.1080/00224499.2023.2176811 36795115 10.1080/00224499.2023.2176811

[CR74] Smolak, L., & Murnen, S. K. (2011). The sexualization of girls and women as a primary antecedent of self-objectification. In R. M. Calogero, S. Tantleff-Dunn, & J. K. Thompson (Eds.), *Self-objectification in women: Causes, consequences, and counteractions* (pp. 53–75). American Psychological Association. 10.1037/12304-003

[CR75] South Palomares, J., & Young, A. (2017). Facial first impressions of partner preference traits: Trustworthiness, status, and attractiveness. *Social Psychological and Personality Science,**9*(8), 990–1000. 10.1177/1948550617732388

[CR76] Thomas, A., Jonason, P. K., Blackburn, J. D., Kennair, L. E. O., Lowe, R., Malouff, J., Stewart-Williams, S., Sulikowski, D., & Li, N. P. (2020). Mate preference priorities in the East and West: A cross-cultural test of the mate preference priority model. *Journal of Personality,**88*(3), 606–620. 10.1111/jopy.12514 31494937 10.1111/jopy.12514

[CR77] Tonidandel, S., LeBreton, J. M., & Johnson, J. W. (2009). Determining the statistical significance of relative weights. *Psychological Methods,**14*(4), 387–399. 10.1037/a0017735 19968399 10.1037/a0017735

[CR78] Travaglia, L. K., Overall, N. C., & Sibley, C. G. (2009). Benevolent and hostile sexism and preferences for romantic partners. *Personality and Individual Differences,**47*(6), 599–604. 10.1016/j.paid.2009.05.015

[CR79] Trivers, R. (1972). Parental investment and sexual selection. In B. Campbell (Ed.), *Sexual selection and the descent of Man* (pp. 136–179). Aldine de Gruyter.

[CR80] Underwood, C. R. (2023). *Multiple approaches to examining gender norms in romantic relationships*. Doctoral dissertation, University of Nevada, Las Vegas. https://digitalscholarship.unlv.edu/cgi/viewcontent.cgi?article=5799&context=thesesdissertations

[CR81] Valentine, K. A., Li, N. P., Meltzer, A. L., & Tsai, M.-H. (2020). Mate preferences for warmth-trustworthiness predict romantic attraction in the early stages of mate selection and satisfaction in ongoing relationships. *Personality and Social Psychology Bulletin,**46*(2), 298–311. 10.1177/0146167219855048 31184259 10.1177/0146167219855048

[CR82] van Anders, S. M. (2015). Beyond sexual orientation: Integrating gender/sex and diverse sexualities via sexual configurations theory. *Archives of Sexual Behavior,**44*(5), 1177–1213. 10.1007/s10508-015-0490-8 25772652 10.1007/s10508-015-0490-8

[CR83] Van Kampen, A., Phillips, M., & Devenport, S. (2024). Young women’s conceptualisation and self-representation in online dating: A qualitative analysis. *SN Social Sciences,**4*(11). 10.1007/s43545-024-00996-5

[CR84] Vares, T. (2018). ‘My [asexuality] is playing hell with my dating life’: Romantic identified asexuals negotiate the dating game. *Sexualities,**21*(4), 520–536. 10.1177/1363460717716400

[CR85] Ward, L. M. (2016). Media and sexualization: State of empirical research, 1995–2015. *Journal of Sex Research,**53*(4–5), 560–577. 10.1080/00224499.2016.1142496 26979592 10.1080/00224499.2016.1142496

[CR86] Williams, S. L., Job, S. A., Todd, E., & Braun, K. (2020). A critical deconstructed quantitative analysis: Sexual and gender minority stress through an intersectional lens. *Journal of Social Issues,**76*(4), 859–879. 10.1111/josi.12410

[CR87] Williams, M., & Sulikowski, D. (2020). Implicit and explicit compromises in long-term partner choice. *Personality and Individual Differences,**166*, Article 110226. 10.1016/j.paid.2020.110226

[CR88] Wilson, T., Temple, J., Lyons, A., & Shalley, F. (2020). What is the size of Australia’s sexual minority population? *BioMedCentral Research Notes,**13*, 1–6. 10.1186/s13104-020-05383-w 10.1186/s13104-020-05383-w PMC7670686 33198795

[CR89] Wood, D., & Brumbaugh, C. (2009). Using revealed mate preferences to evaluate market force and differential preference explanations for mate selection. *Journal of Personality and Social Psychology,**96*(6), 1226–1244. 10.1037/a0015300 19469598 10.1037/a0015300

[CR90] Wood, D., & Furr, R. M. (2016). The correlates of similarity estimates are often misleadingly positive: The nature and scope of the problem, and some solutions. *Personality and Social Psychology Review,**20*(2), 79–99. 10.1177/1088868315581119 25896284 10.1177/1088868315581119 PMC4615254

[CR91] Wu, C. (1983). On the convergence properties of the EM algorithm. *The Annals of Statistics,**11*, 95–103. 10.1214/aos/1176346060

[CR92] Wu, A. K., Marks, M. J., Young, T. M., & Beasley, M. A. (2019). Predictors of bisexual individuals’ dating decisions. *Sexuality and Culture,**24*, 596–612. 10.1007/s12119-019-09651-1

