# Supplementary material for: Diverse Gender and Sexual Identity in Romantic Partner Selection Experiences: An Exploration of Similarities, Differences, and Potential Explanations
Source: Arch Sex Behav. 2025 Jul 15;54(6):2127–49. doi: 10.1007/s10508-025-03173-8 (PMC12283845; doi:10.1007/s10508-025-03173-8)
Supplement: Supplementary file 1 — Supplementary file1 (PDF 431 kb) [file 10508_2025_3173_MOESM1_ESM.pdf]

Appendix A

Table A1

Multiple regression analysis results predicting all test variables except self-perceived mate value with gender and sexual identity, partner trait ideal-ratings, and self-perceived mate value (N = 549).

		β 95% CI					β 95% CI		
	β	Low	High	sr^2		β	Low	High	sr^2
It is challenging to find a romantic partner (Adj. R^2 = .09)					It is challenging to choose a romantic partner (Adj. R^2 = n.s)				
Female	-.03	-.12	.06	.00	Female	.09	-.01	.18	.01
Binary-trans	.03	-.06	.11	.00	Binary-trans	.02	-.07	.11	.00
Non-binary	.04	-.05	.13	.00	Non-binary	-.05	-.14	.04	.00
Same-attracted	.09*	.00	.18	.01	Same-attracted	.04	-.15	.13	.00
Multi-attracted	-.02	-.12	.08	.00	Multi-attracted	.02	-.08	.12	.00
Asexual	.01	-.08	.10	.00	Asexual	.05	-.05	.14	.00
Polyamorous	.03	-.06	.12	.00	Polyamorous	.02	-.07	.11	.00
Ideal-Warm	-.03	-.13	.08	.00	Ideal-Warm	.06	-.05	.17	.00
Ideal-Empowered	-.08	-.18	.02	.00	Ideal-Empowered	.09	-.02	.19	.01
Ideal-Smart	.11*	.02	.20	.01	Ideal-Smart	.00	-.10	.09	.00
Ideal-Dependable	.12*	.02	.22	.01	Ideal-Dependable	-.08	-.18	.03	.00
Ideal-Aesthetic	-.01	-.10	.08	.00	Ideal-Aesthetic	.05	-.05	.14	.00
Ideal-Intimate	.02	-.08	.11	.00	Ideal-Intimate	-.01	-.11	.09	.00
Ideal-Easy-going	-.06	-.15	.03	.00	Ideal-Easy-going	-.02	-.11	.07	.00
Self-Mate-Value	-.26***	-.35	-.17	.06	Self-Mate-Value	-.09	-.18	.01	.01
I quickly decide if a person is suitable as a potential romantic partner (Adj. R^2 = .08)					I put a lot of thought into selecting a romantic partner (Adj. R^2 = n.s)				
Female	.09	-.01	.18	.01	Female	-.03	-.12	.07	.00
Binary-trans	-.05	-.13	.03	.00	Binary-trans	.03	-.06	.12	.00
Non-binary	.00	-.00	.00	.00	Non-binary	.09	-.00	.18	.01
Same-attracted	-.02	-.11	.13	.00	Same-attracted	.00	-.09	.08	.00
Multi-attracted	.01	-.09	.11	.00	Multi-attracted	.01	-.10	.11	.00
Asexual	-.07	-.16	.01	.00	Asexual	.05	-.04	.14	.00
Polyamorous	-.03	-.11	.06	.00	Polyamorous	.01	-.09	.10	.00
Ideal-Warm	-.18***	-.29	-.08	.02	Ideal-Warm	.02	-.09	.13	.00
Ideal-Empowered	-.04	-.14	.06	.00	Ideal-Empowered	.07	-.04	.17	.00
Ideal-Smart	.13**	.03	.22	.01	Ideal-Smart	.04	-.06	.14	.00
Ideal-Dependable	.11*	.01	.21	.01	Ideal-Dependable	.10	-.00	.20	.01
Ideal-Aesthetic	.07	-.02	.16	.00	Ideal-Aesthetic	.01	-.08	.10	.00
Ideal-Intimate	.01	-.08	.10	.00	Ideal-Intimate	-.06	-.15	.04	.00
Ideal-Easy-going	.00	-.08	.08	.00	Ideal-Easy-going	-.02	-.11	.07	.00
Self-Mate-Value	.15***	.06	.24	.02	Self-Mate-Value	.08	-.01	.17	.01

Table A1 continued

		β 95% CI					β 95% CI		
	β	Low	High	sr^2		β	Low	High	sr^2
I believe that people are in different 'leagues' (Adj. R^2 = .05)					I believe that people sometimes 'settle' for a romantic partner (Adj. R^2 = .02)				
Female	.01	-.09	.11	.00	Female	.06	-.04	.16	.00
Binary-trans	-.05	-.13	.04	.00	Binary-trans	-.04	-.13	.05	.00
Non-binary	-.10*	-.19	-.01	.01	Non-binary	-.07	-.16	.03	.00
Same-attracted	-.02	-.12	.09	.00	Same-attracted	.01	-.06	.07	.00
Multi-attracted	.00	-.11	.10	.00	Multi-attracted	.04	-.06	.14	.00
Asexual	.00	-.00	.00	.00	Asexual	.03	-.06	.12	.00
Polyamorous	-.09*	-.18	.00	.01	Polyamorous	.03	-.06	.12	.00
Ideal-Warm	-.08	-.19	.03	.00	Ideal-Warm	-.01	-.12	.10	.00
Ideal-Empowered	-.05	-.15	.05	.00	Ideal-Empowered	-.08	-.18	.02	.00
Ideal-Smart	-.03	-.13	.06	.00	Ideal-Smart	.15**	.05	.25	.02
Ideal-Dependable	.05	-.05	.16	.00	Ideal-Dependable	-.02	-.12	.08	.00
Ideal-Aesthetic	.09	-.01	.18	.01	Ideal-Aesthetic	.02	-.08	.11	.00
Ideal-Intimate	.05	-.04	.15	.00	Ideal-Intimate	.10*	.01	.20	.01
Ideal-Easy-going	.01	-.08	.10	.00	Ideal-Easy-going	.07	-.02	.16	.00
Self-Mate-Value	-.16***	-.25	-.07	.02	Self-Mate-Value	-.02	-.11	.08	.00
I believe in 'love at first sight' (Adj. R^2 = .03)					I believe that sexual identity is fluid (Adj. R^2 = .20)				
Female	-.08	-.17	.02	.00	Female	.15**	.06	.23	.02
Binary-trans	.04	-.05	.12	.00	Binary-trans	.03	-.05	.11	.00
Non-binary	-.06	-.15	.03	.00	Non-binary	.00	-.10	.10	.00
Same-attracted	.08	-.02	.19	.00	Same-attracted	.12**	.04	.22	.01
Multi-attracted	-.02	-.12	.08	.00	Multi-attracted	.30***	.21	.39	.06
Asexual	.00	-.08	.08	.00	Asexual	-.03	-.11	.05	.00
Polyamorous	-.02	-.11	.07	.00	Polyamorous	.05	-.03	.13	.00
Ideal-Warm	-.06	-.17	.05	.00	Ideal-Warm	.16**	.07	.26	.02
Ideal-Empowered	.06	-.04	.16	.00	Ideal-Empowered	-.01	-.11	.09	.00
Ideal-Smart	-.07	-.17	.02	.00	Ideal-Smart	.05	-.04	.13	.00
Ideal-Dependable	.05	-.05	.15	.00	Ideal-Dependable	-.13**	-.23	-.04	.01
Ideal-Aesthetic	.03	-.06	.13	.00	Ideal-Aesthetic	-.06	-.15	.02	.00
Ideal-Intimate	.09	-.01	.19	.01	Ideal-Intimate	-.01	-.11	.08	.00
Ideal-Easy-going	.02	-.07	.11	.00	Ideal-Easy-going	.02	-.06	.10	.00
Self-Mate-Value	.03	-.06	.13	.00	Self-Mate-Value	.03	-.05	.11	.00

Table A1 continued

	β	β 95% CI Low	β 95% CI High	sr^2		β	β 95% CI Low	β 95% CI High	sr^2
Ideal-Warm (Adj. R^2 = .40)					Ideal-Empowered (Adj. R^2 = .33)				
Female	.17***	.09	.24	.02	Female	.08	-.00	.15	.00
Binary-trans	.07*	.00	.14	.00	Binary-trans	-.01	-.08	.06	.00
Non-binary	.01	-.06	.08	.00	Non-binary	.01	-.07	.08	.00
Same-attracted	.12**	.05	.19	.01	Same-attracted	.00	-.04	.04	.00
Multi-attracted	.19***	.12	.27	.03	Multi-attracted	-.07	-.15	.01	.00
Asexual	.08*	.01	.14	.01	Asexual	-.02	-.10	.05	.00
Polyamorous	.10**	.03	.17	.01	Polyamorous	-.01	-.08	.07	.00
Ideal-Warm	-	-	-	-	Ideal-Warm	.08	-.01	.17	.00
Ideal-Empowered	.08	.00	.16	.00	Ideal-Empowered	-	-	-	-
Ideal-Smart	.09*	.01	.16	.01	Ideal-Smart	.32***	.25	.40	.09
Ideal-Dependable	.37***	.30	.45	.11	Ideal-Dependable	.13**	.05	.22	.01
Ideal-Aesthetic	-.12**	-.19	-.04	.01	Ideal-Aesthetic	.07	-.01	.14	.00
Ideal-Intimate	.12**	.04	.19	.01	Ideal-Intimate	.07	-.01	.15	.00
Ideal-Easy-going	.14***	.07	.21	.02	Ideal-Easy-going	.17***	.10	.24	.03
Self-Mate-Value	.02	-.05	.08	.00	Self-Mate-Value	.14***	.07	.21	.02
Ideal-Smart (Adj. R^2 = .22)					Ideal-Dependable (Adj. R^2 = .34)				
Female	.08	-.00	.17	.01	Female	.08	-.00	.16	.01
Binary-trans	-.03	-.11	.05	.00	Binary-trans	.06	-.01	.13	.00
Non-binary	.00	-.11	.11	.00	Non-binary	-.08*	-.16	-.01	.00
Same-attracted	-.04	-.09	.03	.00	Same-attracted	-.05	-.06	.02	.00
Multi-attracted	.01	-.08	.10	.00	Multi-attracted	-.15***	-.23	-.07	.00
Asexual	.01	-.07	.09	.00	Asexual	.00	-.06	.07	.00
Polyamorous	-.08	-.16	.00	.01	Polyamorous	-.13***	-.21	-.06	.01
Ideal-Warm	.11*	.02	.21	.01	Ideal-Warm	.41***	.33	.49	.01
Ideal-Empowered	.38***	.29	.47	.11	Ideal-Empowered	.13**	.05	.21	.11
Ideal-Smart	-	-	-	-	Ideal-Smart	.00	-.09	.08	.00
Ideal-Dependable	.00	-.10	.09	.00	Ideal-Dependable	-	-	-	-
Ideal-Aesthetic	.09*	.00	.17	.01	Ideal-Aesthetic	.03	-.05	.10	.01
Ideal-Intimate	.04	-.04	.13	.00	Ideal-Intimate	.18***	.10	.26	.00
Ideal-Easy-going	-.01	-.09	.07	.00	Ideal-Easy-going	.01	-.07	.09	.00
Self-Mate-Value	-.02	-.09	.06	.00	Self-Mate-Value	-.02	-.10	.05	.00

Table A1 continued

		β 95% CI					β 95% CI		
	β	Low	High	sr^2		β	Low	High	sr^2
Ideal-Aesthetic ($Adj. R^2 = .21$)					Ideal-Intimate ($Adj. R^2 = .26$)				
Female	-.22***	-.31	-.14	.01	Female	-.06	-.14	.03	.00
Binary-trans	-.03	-.11	.05	.00	Binary-trans	-.04	-.12	.03	.00
Non-binary	.01	-.07	.10	.00	Non-binary	-.02	.02	.12	.00
Same-attracted	-.11**	-.15	-.02	.01	Same-attracted	.12**	-.05	.13	.01
Multi-attracted	-.13**	-.22	-.04	.01	Multi-attracted	.07	-.02	.16	.00
Asexual	.01	-.07	.09	.00	Asexual	-.16***	-.24	-.09	.02
Polyamorous	-.04	-.13	.04	.01	Polyamorous	.12**	.04	.19	.01
Ideal-Warm	-.15**	-.25	-.06	.01	Ideal-Warm	.14**	.05	.24	.01
Ideal-Empowered	.08	-.02	.17	.11	Ideal-Empowered	.08	-.01	.17	.00
Ideal-Smart	.09*	.00	.17	.00	Ideal-Smart	.04	-.04	.12	.00
Ideal-Dependable	.03	-.06	.13	.00	Ideal-Dependable	.20***	.12	.29	.03
Ideal-Aesthetic	-	-	-	-	Ideal-Aesthetic	.25***	.17	.33	.05
Ideal-Intimate	.26***	.18	.35	.05	Ideal-Intimate	-	-	-	-
Ideal-Easy-going	-.03	-.11	.05	.00	Ideal-Easy-going	.07	-.01	.14	.00
Self-Mate-Value	.14***	.06	.22	.02	Self-Mate-Value	.05	-.03	.13	.00
Ideal-Easy-going ($Adj. R^2 = .12$)					Self-Warm ($Adj. R^2 = .34$)				
Female	-.13**	-.22	-.04	.01	Female	-.06	-.14	.02	.00
Binary-trans	-.10*	-.18	-.02	.01	Binary-trans	.01	-.06	.09	.00
Non-binary	.05	-.04	.14	.00	Non-binary	.03	-.05	.11	.00
Same-attracted	-.08	-.10	.01	.00	Same-attracted	.01	-.03	.05	.00
Multi-attracted	-.06	-.15	.04	.00	Multi-attracted	.02	-.07	.11	.00
Asexual	-.01	-.09	.07	.00	Asexual	.05	-.02	.12	.00
Polyamorous	-.04	-.13	.04	.00	Polyamorous	.01	-.06	.08	.00
Ideal-Warm	.21***	.10	.31	.03	Ideal-Warm	.50***	.41	.59	.14
Ideal-Empowered	.22***	.13	.32	.03	Ideal-Empowered	-.06	-.14	.03	.00
Ideal-Smart	-.02	-.10	.08	.00	Ideal-Smart	.02	-.06	.10	.00
Ideal-Dependable	.01	-.08	.11	.00	Ideal-Dependable	.02	-.07	.11	.00
Ideal-Aesthetic	-.03	-.12	.06	.00	Ideal-Aesthetic	-.13**	-.20	-.05	.01
Ideal-Intimate	.08	-.01	.17	.00	Ideal-Intimate	.13**	.05	.21	.01
Ideal-Easy-going	-	-	-	-	Ideal-Easy-going	.07	-.00	.14	.00
Self-Mate-Value	-.01	-.09	.07	.00	Self-Mate-Value	.12**	.05	.19	.01

Table A1 continued

	β	β 95% CI		sr^2		β	β 95% CI		sr^2
		Low	High				Low	High	
Self-Empowered (Adj. R^2 = .37)					Self-Smart (Adj. R^2 = .21)				
Female	-.10*	-.17	-.02	.01	Female	-.09*	-.18	.00	.01
Binary-trans	-.02	-.09	.05	.00	Binary-trans	.00	-.00	.00	.00
Non-binary	.01	-.06	.08	.00	Non-binary	.01	-.07	.09	.00
Same-attracted	.00	-.04	.05	.00	Same-attracted	.07	-.01	.09	.00
Multi-attracted	-.02	-.11	.06	.00	Multi-attracted	.03	-.06	.12	.00
Asexual	.00	-.08	.08	.00	Asexual	.00	-.11	.10	.00
Polyamorous	-.03	-.10	.04	.00	Polyamorous	-.01	-.09	.06	.00
Ideal-Warm	-.01	-.09	.08	.00	Ideal-Warm	.12*	.02	.22	.01
Ideal-Empowered	.39***	.31	.47	.10	Ideal-Empowered	-.21***	-.30	-.11	.03
Ideal-Smart	-.01	-.08	.06	.00	Ideal-Smart	.45***	.37	.54	.16
Ideal-Dependable	.06	-.03	.14	.00	Ideal-Dependable	.03	-.07	.12	.00
Ideal-Aesthetic	.00	-.08	.09	.00	Ideal-Aesthetic	.02	-.06	.10	.00
Ideal-Intimate	.00	-.08	.07	.00	Ideal-Intimate	.00	-.12	.11	.00
Ideal-Easy-going	.07	-.01	.14	.00	Ideal-Easy-going	-.01	-.09	.07	.00
Self-Mate-Value	.36***	.29	.43	.11	Self-Mate-Value	.20***	.12	.28	.03
Self-Dependable (Adj. R^2 = .33)					Self-Aesthetic (Adj. R^2 = .41)				
Female	-.06	-.14	.02	.00	Female	-.02	-.09	.06	.00
Binary-trans	.03	-.04	.11	.00	Binary-trans	.05	-.02	.12	.00
Non-binary	.00	-.00	.00	.00	Non-binary	.04	-.03	.11	.00
Same-attracted	-.05	-.07	.02	.00	Same-attracted	-.04	-.09	.02	.00
Multi-attracted	-.04	-.13	.04	.00	Multi-attracted	.03	-.06	.11	.00
Asexual	.02	-.05	.10	.00	Asexual	.03	-.04	.10	.00
Polyamorous	-.02	-.10	.05	.00	Polyamorous	-.06	-.13	.01	.00
Ideal-Warm	.07	-.02	.16	.00	Ideal-Warm	.00	-.07	.08	.00
Ideal-Empowered	.04	-.05	.12	.00	Ideal-Empowered	.07	-.01	.15	.00
Ideal-Smart	.03	-.05	.11	.00	Ideal-Smart	.03	-.04	.10	.00
Ideal-Dependable	.52***	.43	.60	.17	Ideal-Dependable	-.05	-.13	.04	.00
Ideal-Aesthetic	-.13**	-.21	-.05	.01	Ideal-Aesthetic	.31***	.23	.38	.07
Ideal-Intimate	-.01	-.09	.07	.00	Ideal-Intimate	.04	-.04	.11	.00
Ideal-Easy-going	.05	-.03	.12	.00	Ideal-Easy-going	.02	-.05	.08	.00
Self-Mate-Value	.04	-.03	.12	.00	Self-Mate-Value	.49***	.42	.56	.20

Table A1 continued

	β	β 95% CI Low	β 95% CI High	sr^2		β	β 95% CI Low	β 95% CI High	sr^2
Self-Intimate (Adj. R^2 = .51)					Self- Easygoing (Adj. R^2 = .29)				
Female	-.07	-.13	.00	.00	Female	-.25	-.33	-.17	.05
Binary-trans	.00	-.05	.06	.00	Binary-trans	-.03	-.10	.05	.00
Non-binary	.04	-.02	.11	.00	Non-binary	-.02	-.10	.06	.00
Same-attracted	.01	-.04	.05	.00	Same-attracted	-.07	-.10	.01	.00
Multi-attracted	.04	-.03	.11	.00	Multi-attracted	-.03	-.12	.06	.00
Asexual	-.03	-.09	.03	.00	Asexual	.01	-.06	.09	.00
Polyamorous	.04	-.02	.10	.00	Polyamorous	.00	-.12	.11	.00
Ideal-Warm	.02	-.06	.10	.00	Ideal-Warm	-.06	-.15	.03	.00
Ideal-Empowered	.01	-.06	.09	.00	Ideal-Empowered	-.07	-.16	.01	.00
Ideal-Smart	-.02	-.09	.04	.00	Ideal-Smart	-.04	-.12	.04	.00
Ideal-Dependable	.05	-.03	.12	.00	Ideal-Dependable	.10*	.02	.19	.01
Ideal-Aesthetic	-.06	-.13	.01	.00	Ideal-Aesthetic	-.04	-.12	.04	.00
Ideal-Intimate	.65***	.58	.72	.30	Ideal-Intimate	.06	-.02	.14	.00
Ideal-Easy-going	-.01	-.07	.06	.00	Ideal-Easy-going	.47***	.39	.54	.19
Self-Mate-Value	.19***	.13	.26	.03	Self-Mate-Value	.11**	.04	.19	.01

Note. *$p < .05$, ** $p < .01$, *** $p < .001$. sr^2 = semi-partial correlation. CI = Confidence Interval.
Adj. R^2 = Significant Adjusted R^2. n.s = Non-significant.

Appendix B

Table B1

Multiple regression analysis results predicting self-perceived mate value with gender and sexual identity, and partner trait self-ratings for all subsamples (N = 549).

		β 95% CI					β 95% CI		
	β	Low	High	sr^2		β	Low	High	sr^2
Overall (n = 549, Adj. R^2 = .43)					Different-attracted (n = 283, Adj. R^2 = .45)				
Female	.17***	.10	.24	.02	Female	.18***	.09	.28	.03
Binary-trans	-.04	-.10	.03	.00	Binary-trans[a]	-.04	-.13	.05	.00
Non-binary	-.05	-.12	.02	.00	Non-binary[a]	-.04	-.13	.05	.00
Same-attracted	.03	-.04	.10	.00	Same-attracted	-	-	-	-
Multi-attracted	-.05	-.13	.02	.00	Multi-attracted	-	-	-	-
Asexual	-.12***	-.19	-.05	.01	Asexual	-.09*	-.18	00	.01
Polyamorous	.08*	.01	.14	.01	Polyamorous[a]	-.03	-.12	.03	.00
Self-Warm	.06	-.02	.14	.00	Self-Warm	.04	-.07	.15	.00
Self-Empowered	.26***	.18	.33	.04	Self-Empowered	.28***	.18	.39	.05
Self-Smart	-.01	-.07	.06	.00	Self-Smart	-.03	-.13	.06	.00
Self-Dependable	-.06	-.14	.01	.00	Self-Dependable	.00	-.10	.10	.00
Self-Aesthetic	.43***	.36	.50	.15	Self-Aesthetic	.47***	.37	.56	.18
Self-Intimate	.07	.00	.15	.00	Self-Intimate	.03	-.08	.14	.00
Self-Easy-going	.02	-.05	.09	.00	Self-Easy-going	.08	-.02	.17	.00
Same-attracted (n = 99, Adj. R^2 = .35)					Multi-attracted (n = 167, Adj. R^2 = .43)				
Female	.19	-.00	.38	.03	Female	.16*	.02	.29	.02
Binary-trans[a]	-.10	-.28	.08	.02	Binary-trans	.00	-.11	.12	.00
Non-binary	-.18	-.38	.02	.00	Non-binary	-.06	-.18	.06	.00
Same-attracted	-	-	-	-	Same-attracted	-	-	-	-
Multi-attracted	-	-	-	-	Multi-attracted	-	-	-	-
Asexual[a]	.02	-.17	.20	.00	Asexual	-.18**	-.31	-.05	.03
Polyamorous[a]	.14	-.05	.33	.01	Polyamorous	.12	-.00	.24	.01
Self-Warm	-.05	-.34	.24	.00	Self-Warm	.08	-.06	.22	.00
Self-Empowered	.26*	.06	.46	.04	Self-Empowered	.21**	.06	.36	.03
Self-Smart	.15	-.05	.35	.01	Self-Smart	.00	-.23	.23	.00
Self-Dependable	-.20	-.44	.03	.02	Self-Dependable	-.06	-.19	.08	.00
Self-Aesthetic	.41***	.20	.61	.10	Self-Aesthetic	.41***	.28	.55	.13
Self-Intimate	.02	-.21	.24	.00	Self-Intimate	.15*	.01	.28	.02
Self-Easy-going	.09	-.11	.28	.00	Self-Easy-going	-.04	-.17	.10	.00

Table B1 continued

		β 95% CI					β 95% CI		
	β	Low	High	*sr*²		β	Low	High	*sr*²
Male (n = 225, Adj. R^2 = .43)					Female (n = 324, Adj. R^2 = .43)				
Female	-	-	-	-	Female	-	-	-	-
Binary-trans	-.06	-.18	.05	.00	Binary-trans	-.03	-.12	.05	.00
Non-binary[a]	-.12*	-.23	-.01	.01	Non-binary	-.02	-.12	.07	.00
Same-attracted	.06	-.05	.17	.00	Same-attracted	-.02	-.11	.08	.00
Multi-attracted	.01	-.11	.12	.00	Multi-attracted	-.08	-.18	.01	.01
Asexual	-.10	-.20	.01	.01	Asexual	-.15***	-.24	-.06	.02
Polyamorous	.09	-.02	.20	.01	Polyamorous	.08	-.01	.17	.01
Self-Warm	.06	-.07	.19	.00	Self-Warm	.07	-.04	.18	.00
Self-Empowered	.34***	.22	.46	.08	Self-Empowered	.20***	10	.30	.03
Self-Smart	-.03	-.14	.08	.00	Self-Smart	.00	-.09	.09	.00
Self-Dependable	.01	-.11	.13	.00	Self-Dependable	-.11*	-.21	-.01	.01
Self-Aesthetic	.47***	.35	.58	.16	Self-Aesthetic	.42***	.33	.51	.14
Self-Intimate	-.06	-.18	.07	.00	Self-Intimate	.16**	.06	.26	.02
Self-Easy-going	.04	-.07	.15	.00	Self-Easy-going	.02	-.08	.11	.00
Man (n = 217, Adj. R^2 = .45)					Woman (n = 288, Adj. R^2 = .43)				
Female	-	-	-	-	Female	-	-	-	-
Binary-trans	.04	-.07	.15	.00	Binary-trans	-.09*	-.18	-.00	.01
Non-binary	-	-	-	-	Non-binary	-	-	-	-
Same-attracted	.09	-.01	.20	.01	Same-attracted	-.05	-.15	.04	.00
Multi-attracted	.02	-.09	.12	.00	Multi-attracted	-.07	-.17	.03	.00
Asexual	-.13*	-.23	-.02	.01	Asexual	-.13**	-.23	-.04	.02
Polyamorous	-.01	-.11	.09	.00	Polyamorous	.01	-.08	.10	.00
Self-Warm	.02	-.11	.16	.00	Self-Warm	.08	-.03	.19	.00
Self-Empowered	.29***	.17	.40	.06	Self-Empowered	.23***	.12	.34	.03
Self-Smart	.01	-.10	.12	.00	Self-Smart	-.02	-.12	.07	.00
Self-Dependable	.02	-.10	.14	.00	Self-Dependable	-.11*	-.21	-.01	.01
Self-Aesthetic	.50***	.38	.61	.18	Self-Aesthetic	.41***	.31	.51	.13
Self-Intimate	-.01	-.14	.13	.00	Self-Intimate	.12*	.02	.23	.01
Self-Easy-going	.04	-.07	.15	.00	Self-Easy-going	.06	-.03	.16	.00

Table B1 continued

	β	β 95% CI Low	β 95% CI High	sr^2		β	β 95% CI Low	β 95% CI High	sr^2
Binary (n = 505, Adj. R^2 = .45)					Non-binary (n = 44, Adj. R^2 = .40)†				
Female	.16***	.09	.23	.02	Female[a]	.38*	.06	.70	.08
Binary-trans	-.03	-.10	.03	.00	Binary-trans	-	-	-	-
Non-binary	-	-	-	-	Non-binary	-	-	-	-
Same-attracted	.03	-.04	.10	.00	Same-attracted[a]	-.41	-1.30	.48	.01
Multi-attracted	-.03	-.10	.05	.00	Multi-attracted[a]	-.46	-1.37	.45	.02
Asexual	-.13***	-.20	-.06	.00	Asexual	-.12	-.44	.20	.01
Polyamorous	-.01	-.08	.06	.02	Polyamorous	.52***	.24	.80	.20
Self-Warm	.05	-.04	.13	.00	Self-Warm	.10	-.25	.46	.00
Self-Empowered	.25***	.17	.33	.06	Self-Empowered	.25	-.10	.60	.03
Self-Smart	-.01	-.07	.06	.00	Self-Smart	.07	-.24	.39	.00
Self-Dependable	.05	-.12	.03	.00	Self-Dependable	-.09	-.40	.22	.00
Self-Aesthetic	.44***	.37	.52	.20	Self-Aesthetic	.27	-.01	.55	.05
Self-Intimate	.07	-.01	.15	.01	Self-Intimate	.19	-.10	.48	.02
Self-Easy-going	.05	-.02	.12	.00	Self-Easy-going	-.05	-.38	.28	.00
Binary-trans (n = 27, Adj. R^2 = .52)†					TGD (n = 71, Adj. R^2 = .35)				
Female	.24	-.12	.60	.04	Female	.19	-.02	.40	.03
Binary-trans	-	-	-	-	Binary-trans	-	-	-	-
Non-binary	-	-	-	-	Non-binary	-	-	-	-
Same-attracted[a]	-.11	-.69	.48	.00	Same-attracted[a]	-.01	-.41	.39	.00
Multi-attracted[a]	.14	-.45	.73	.00	Multi-attracted[a]	.01	-.39	.42	.00
Asexual[a]	-.44*	-.83	-.05	.11	Asexual	-.18	-.41	.06	.02
Polyamorous[a]	-.25	-.65	.15	.03	Polyamorous	.28*	.06	.50	.06
Self-Warm	.23	-.19	.64	.03	Self-Warm	.12	-.13	.37	.01
Self-Empowered	.66**	.26	1.06	.23	Self-Empowered	.34**	.09	.60	.07
Self-Smart	-.07	-.47	.33	.00	Self-Smart	.07	-.15	.30	.00
Self-Dependable	-.10	-.57	.38	.00	Self-Dependable	-.05	-.29	.19	.00
Self-Aesthetic	-.02	-.45	.42	.00	Self-Aesthetic	.32**	.10	.54	.08
Self-Intimate	.24	-.16	.65	.03	Self-Intimate	.12	-.11	.34	.01
Self-Easy-going	-.37*	-.73	-.01	.09	Self-Easy-going	-.25*	-.48	-.03	.05

Table B1 continued

	β	β 95% CI		sr^2		β	β 95% CI		sr^2
		Low	High				Low	High	
Bisexual<br>(*n* = 114, Adj. R^2 = .44)					Pansexual<br>(*n* = 35, Adj. R^2 = .55)†				
Female	.14	-.02	.30	.01	Female[a]	.18	-.12	.47	.02
Binary-trans	.06	-.09	.22	.00	Binary-trans[a]	-.10	-.40	.20	.01
Non-binary	-.01	-.16	.14	.00	Non-binary	-.35*	-.61	-.09	.10
Same-attracted	-	-	-	-	Same-attracted	-	-	-	-
Multi-attracted	-	-	-	-	Multi-attracted	-	-	-	-
Asexual[a]	.00	-.00	.00	.00	Asexual[a]	-.26	-.55	.03	.04
Polyamorous	.08	-.08	.23	.00	Polyamorous[a]	.36*	.05	.67	.08
Self-Warm	.08	-.08	.25	.01	Self-Warm	.23	-.08	.54	.03
Self-Empowered	.23*	.05	.42	.03	Self-Empowered	.58**	.22	.93	.15
Self-Smart	.08	-.07	.23	.01	Self-Smart	-.09	-.43	.26	.00
Self-Dependable	-.09	-.25	.07	.01	Self-Dependable	.09	-.19	.37	.01
Self-Aesthetic	.43***	.27	.59	.14	Self-Aesthetic	.23	-.07	.53	.03
Self-Intimate	.22*	.05	.38	.03	Self-Intimate	-.10	-.43	.24	.00
Self-Easy-going	-.02	-.18	.13	.00	Self-Easy-going	-.06	-.40	.29	.00
Allosexual<br>(*n* = 511, Adj. R^2 = .40)					Asexual<br>(*n* = 38, Adj. R^2 = .38)†				
Female	.19***	.12	.26	.03	Female	.11	-.32	.54	.00
Binary-trans	-.03	-.10	.05	.00	Binary-trans[a]	-.18	-.49	.13	.02
Non-binary	-.08*	-.15	.00	.01	Non-binary	-.04	-.38	.30	.00
Same-attracted	.03	-.04	.10	.00	Same-attracted[a]	-.07	-.44	.30	.00
Multi-attracted	-.03	-.11	.05	.00	Multi-attracted	-.22	-.62	.17	.02
Asexual	-	-	-	-	Asexual	-	-	-	-
Polyamorous	.07	-.00	.14	.00	Polyamorous[a]	.04	-.30	.38	.00
Self-Warm	.02	-.07	.10	.00	Self-Warm	.31	-.06	.67	.05
Self-Empowered	.29***	.20	.37	.06	Self-Empowered	.17	-.23	.56	.01
Self-Smart	-.02	-.09	.06	.00	Self-Smart	-.09	-.45	.28	.00
Self-Dependable	-.04	-.12	.04	.00	Self-Dependable	-.11	-.46	.24	.01
Self-Aesthetic	.42***	.34	.50	.14	Self-Aesthetic	.52**	.21	.83	.20
Self-Intimate	.06	-.02	.14	.00	Self-Intimate	.21	-.18	.60	.02
Self-Easy-going	.04	-.04	.10	.00	Self-Easy-going	-.09	-.47	.29	.00

Table B1 continued

	β	β 95% CI Low	β 95% CI High	sr^2		β	β 95% CI Low	β 95% CI High	sr^2
Monoamorous (n = 512, Adj. R^2 = .44)					Polyamorous (n = 37, Adj. R^2 = .59)✝				
Female	.18⁂	.11	.25	.03	Female	-.04	-.33	.25	.00
Binary-trans	-.02	-.09	.05	.00	Binary-trans	-.11	-.35	.14	.03
Non-binary	-.11⁑	-.18	-.04	.01	Non-binary	.23	-.09	.55	.03
Same-attracted	.03	-.05	.10	.00	Same-attracted[a]	.25	-.10	.59	.02
Multi-attracted	-.05	-.12	.03	.00	Multi-attracted[a]	.18	-.15	.51	.01
Asexual	-.11⁑	-.18	-.04	.01	Asexual[a]	-.26	-.54	.01	.04
Polyamorous	-	-	-	-	Polyamorous	-	-	-	-
Self-Warm	.04	-.05	.12	.00	Self-Warm	.26	-.01	.53	.05
Self-Empowered	.25⁂	.17	.33	.04	Self-Empowered	.14	-.15	.43	.01
Self-Smart	.01	-.06	.08	.00	Self-Smart	-.03	-.34	.29	.00
Self-Dependable	-.06	-.13	.02	.00	Self-Dependable	-.05	-.32	.23	.00
Self-Aesthetic	.41⁂	.34	.49	.13	Self-Aesthetic	.70⁂	.42	.98	.30
Self-Intimate	.09*	.01	.17	.01	Self-Intimate	-.13	-.43	.17	.01
Self-Easy-going	.04	-.03	.11	.00	Self-Easy-going	-.12	-.43	.18	.01
Majority (n = 261, Adj. R^2 = .44)					Minority (n = 288, Adj. R^2 = .42)				
Female	.22⁂	.12	.32	.04	Female	.13*	.02	.23	.01
Binary-trans	-	-	-	-	Binary-trans	-.04	-.13	.05	.00
Non-binary	-	-	-	-	Non-binary	-.07	-.16	.03	.00
Same-attracted	-	-	-	-	Same-attracted	.06	-.14	.25	.00
Multi-attracted	-	-	-	-	Multi-attracted	-.02	-.21	.18	.00
Asexual	-	-	-	-	Asexual	-.13*	-.24	-.03	.01
Polyamorous	-	-	-	-	Polyamorous	.09	-.01	.18	.01
Self-Warm	-.03	-.15	.10	.00	Self-Warm	.12*	.00	.23	.01
Self-Empowered	.29⁂	.18	.41	.06	Self-Empowered	.24⁂	.13	.35	.04
Self-Smart	-.05	-.15	.05	.00	Self-Smart	.04	-.06	.14	.00
Self-Dependable	.03	-.08	.14	.00	Self-Dependable	-.13*	-.23	-.03	.01
Self-Aesthetic	.45⁂	.35	.55	.17	Self-Aesthetic	.42⁂	.32	.52	.13
Self-Intimate	.04	-.07	.16	.00	Self-Intimate	.09	-.01	.20	.01
Self-Easy-going	.09	-.01	.19	.01	Self-Easy-going	-.03	-.12	.07	.00

Note. *p < .05, ⁑ p < .01, ⁂ p < .001. sr^2 = semi-partial correlation. CI = Confidence Interval. Adj. R^2 = Adjusted R^2. TGD = Trans and gender diverse.

[a] = Interpret this predictor with caution as there a low subsample sizes (n < 10) in one or both categories.

✝ = Interpret with caution as subsample is underpowered when considering expected effect size (adjusted R^2 = .45, Csajbók and Berkics, 2017) and low sample size (n < 66).
